# Genome-wide analysis of alternative splicing in Chlamydomonas reinhardtii

**DOI:** 10.1186/1471-2164-11-114

**Published:** 2010-02-17

**Authors:** Adam Labadorf, Alicia Link, Mark F Rogers, Julie Thomas, Anireddy SN Reddy, Asa Ben-Hur

**Affiliations:** 1 Computer Science Department, Colorado State University, Fort Collins, CO, USA; 2 Department of Biology and Program in Molecular Plant Biology, Colorado State University, Fort Collins, CO, USA

## Abstract

**Background:**

Genome-wide computational analysis of alternative splicing (AS) in several flowering plants has revealed that pre-mRNAs from about 30% of genes undergo AS. *Chlamydomonas*, a simple unicellular green alga, is part of the lineage that includes land plants. However, it diverged from land plants about one billion years ago. Hence, it serves as a good model system to study alternative splicing in early photosynthetic eukaryotes, to obtain insights into the evolution of this process in plants, and to compare splicing in simple unicellular photosynthetic and non-photosynthetic eukaryotes. We performed a global analysis of alternative splicing in *Chlamydomonas reinhardtii *using its recently completed genome sequence and all available ESTs and cDNAs.

**Results:**

Our analysis of AS using BLAT and a modified version of the Sircah tool revealed AS of 498 transcriptional units with 611 events, representing about 3% of the total number of genes. As in land plants, intron retention is the most prevalent form of AS. Retained introns and skipped exons tend to be shorter than their counterparts in constitutively spliced genes. The splice site signals in all types of AS events are weaker than those in constitutively spliced genes. Furthermore, in alternatively spliced genes, the prevalent splice form has a stronger splice site signal than the non-prevalent form. Analysis of constitutively spliced introns revealed an over-abundance of motifs with simple repetitive elements in comparison to introns involved in intron retention. In almost all cases, AS results in a truncated ORF, leading to a coding sequence that is around 50% shorter than the prevalent splice form. Using RT-PCR we verified AS of two genes and show that they produce more isoforms than indicated by EST data. All cDNA/EST alignments and splice graphs are provided in a website at http://combi.cs.colostate.edu/as/chlamy.

**Conclusions:**

The extent of AS in *Chlamydomonas *that we observed is much smaller than observed in land plants, but is much higher than in simple unicellular heterotrophic eukaryotes. The percentage of different alternative splicing events is similar to flowering plants. Prevalence of constitutive and alternative splicing in *Chlamydomonas*, together with its simplicity, many available public resources, and well developed genetic and molecular tools for this organism make it an excellent model system to elucidate the mechanisms involved in regulated splicing in photosynthetic eukaryotes.

## Background

The coding regions (exons) of most eukaryotic genes are interrupted by non-coding sequences (introns). The intronic sequences from primary transcripts (also called precursor-mRNAs or pre-mRNAs) are removed and the exons are spliced to generate functional mRNAs [1]. In many organisms, pre-mRNAs are alternatively spliced to generate multiple mRNAs from a single gene [1]. It is becoming clear that alternative splicing generates distinct proteins with altered functions from a limited set of genes [2-5]. The effects of alternative splicing on proteins include production of protein isoforms with loss or gain of function, altered subcellular localization, protein stability and/or posttranslational modifications [1,3]. Furthermore, alternative splicing plays a role in regulation of gene expression through processes such as regulated unproductive splicing and translation (RUST) and mRNA recruitment [6,7]. Alternative splicing is also implicated in evolution of organisms [8]. The availability of the complete genome sequences of many multicellular eukaryotic organisms and large sets of full-length cDNAs and ESTs has permitted comprehensive analysis of alternative splicing. More recently, global analysis of alternative splicing has also been performed using splicing sensitive mircroarrays and new generation sequencing technologies [5,8,9]. These analyses have shown that alternative splicing is highly prevalent in multicellular eukaryotes. In humans, 95% of multiexon genes undergo alternative splicing resulting in the generation of two or more transcripts from a single gene [5]. Analysis of alternative splicing in flowering plants by aligning the available cDNAs/ESTs to genome sequences has shown that pre-mRNAs from *~ *30% of genes are alternatively spliced [10,11]. Alternative splicing in some specific gene families such as genes encoding serine/arginine-rich proteins is extensive, resulting in a five-fold increase in transcriptome complexity [12,13]. In addition, stresses have been shown to dramatically alter the splicing pattern of many plant genes [3,12-16]. In mammalian systems, exon skipping is most prevalent, whereas in flowering plants up to 55% of alternative splicing events are intron retention [3,10,11,17]. It is suggested that the variations in frequencies of different types of alternative splicing events between plant and non-plant systems reflect the differences in gene architecture and pre-mRNA splicing between these organisms [3,11]. In contrast to multicellular organisms, very little is known about the prevalence and types of alternative splicing in simple unicellular photosynthetic eukaryotes from which land plants have evolved. Recent completion of the *Chlamydomonas *genome and the availability of a fairly large number of ESTs [18-20] permit global analysis of post-transcriptional events including alternative splicing in a unicellular photosynthetic eukaryote.

*Chlamydomonas *shares many features with cells of more complex eukaryotic plants and animals. *Chlamydomonas*, like land plants, is an autotroph and contains a chloroplast. Furthermore, like animals, it can grow as a heterotroph and is mobile. *Chlamydomonas *diverged from land plants about one billion years ago [18]. About 93% of the 120 Mb genome of *Chlamydomonas reinhardtii *has been sequenced [18,19]. Gene models in the latest version (v4) of the *Chlamydomonas *genome sequence predict 16,709 protein-coding genes and about half of these gene predictions have cDNA/EST support. Analysis of the *Chlamydomonas *genome revealed that it contains many genes that are specific to both plant and animal lineages, reflecting its unique position in evolution [18]. Because of the many advantages *Chlamydomonas *offers, it is considered to be the "green yeast" for studying various eukaryotic cellular processes [21]. For over five decades, *Chlamydomonas *has been used as a model system to study many aspects of photosynthesis, structure and function of flagella, and a variety of other biological processes. More recently, it is being used to investigate mechanisms that regulate biofuels production [22]. Analysis of alternative splicing in *Chlamydomonas *allows comparison of alternative splicing between simple unicellular photosynthetic eukaryotes and highly evolved flowering plants.

Furthermore, this will also aid in understanding how alternative splicing has evolved during the evolution of land plants. Hence, we have performed a comprehensive analysis of alternative splicing in *Chlamydomonas reinhardtii*. We have used the BLAT tool [23] and a modified version of the Sircah software for the detection and visualization of alternative splicing [24]. Detailed results, including alignments and splice graphs for each cluster exhibiting alternative splicing are available on our *"Chlamydomonas AS" *site http://combi.cs.colostate.edu/as/chlamy/. Our results show that alternative splicing is prevalent in *Chlamydomonas*, although the extent of it is less than in land plants. The relative frequency of different splicing events in *Chlamydomonas *is very similar to higher plants.

## Results and Discussion

### Properties of introns

Unlike other unicellular eukaryotes (e.g. yeast), the vast majority of genes (*~ *88%) in *Chlamydomonas *have introns (see Table S1 in the Additional file 1 for a comparison of the properties of the *Chlamydomonas *genome with humans and Arabidopsis). The percentage of intron-containing genes in *Chlamydomonas *is higher than in plants and humans.

Previous comparative studies on gene structure in flowering plants and animals have revealed a number of significant differences in their gene architecture [25,26]. For example, land plant genes are shorter than animal genes with fewer exons and shorter introns [3].

Furthermore, plant introns are rich in T or T/A, which is necessary for the recognition of splice sites and efficient splicing of pre-mRNAs [25,26]. *Chlamydomonas *shares both plant and animal features in its gene architecture. The average number of introns in *Chlamydomonas *is similar to humans. However, the median size of exons (132 bases) and introns (232 nucleotides) is similar to flowering plants. The GC content of *Chlamydomonas *is 64%, which is significantly higher than the GC content of multi-cellular organisms. Introns in protein coding genes of metazoans have four signals that are necessary for accurate splicing of pre-mRNAs. These include two consensus sequences at the 5' and 3' splice sites with conserved GT and AG dinucleotides, respectively, a polypyrimidine tract at the 3' end of the intron, and a branch point located about 17-40 nucleotides upstream of the 3' splice site [1]. However, in land-plants the branch point is not obvious; the 3' end of plant introns are rich in T nucleotides [25]. Although the 5' and 3' splice sites in *Chlamydomonas *introns are similar to land plants and humans (Figure S5 in Additional file 1) the 3' end of introns in *Chlamydomonas *is enriched in C in place of a polyprimidine tract (Figure S6 in Additional file 1).

### Extent and types of alternative splicing

**Figure 1:**
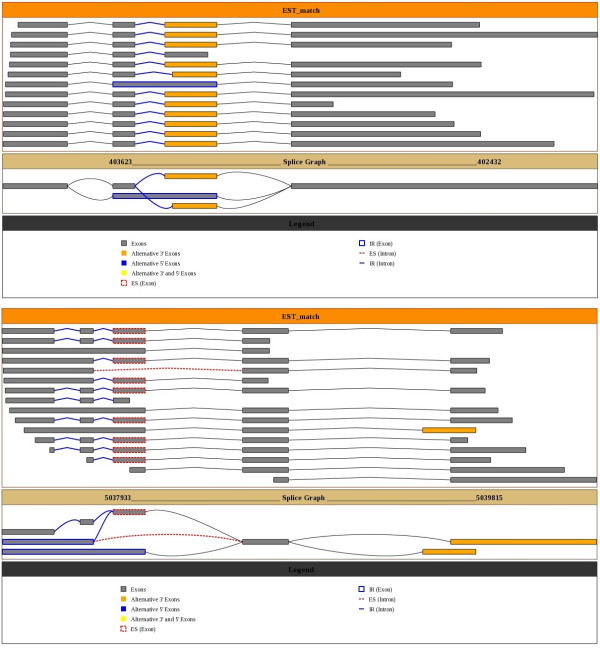
**Example splice graph**. Shown are two splice graphs along with the relevant EST evidence for the gene fgenesh2_pg.C_scaffold_39000087, which exhibits intron retention and Alt3' (top), and for the gene estExt_fgenesh2_kg.C_380020, which exhibits intron retention, exon skipping, and Alt3' (bottom). Figures were generated by Sircah as part of our pipeline.

We developed a pipeline for detection and visualization of alternative splicing in *Chlamydomonas *based on EST-to-genome alignments using BLAT [23], and a modified version of the Sircah alternative splicing detection software [24]. Details are found in the Methods section and Additional file 1. For EST data we used a recently constructed EST dataset containing 252,484 ESTs processed using cDNA termini to anchor transcripts to their correct positions in the genome [20]. ESTs were aligned to the genome and grouped into *clusters *that overlap in their genomic coordinates and occur on the same strand. Our alignment and alternative splicing detection pipeline resulted in 498 clusters that show 611 alternative splicing events. The alternative splicing events in each cluster are summarized with splice graphs [27]. Example splice graphs are shown in Figure 1. A companion website provides visualization of the EST alignments and splice graphs for each cluster showing alternative splicing, as well as access to the alignments themselves and additional information (see http://combi.cs.colostate.edu/as/chlamy/). Of the clusters that show alternative splicing, 484 were associated with predicted genes in version 4.0 of the *Chlamydomonas *genome [28].

We classified all observed alternative splicing events into the following five groups: Intron Retention (IR), Alternative 5' splice site (Alt5'), Alternative 3' splice site (Alt3'), events where both the 5' and the 3' end of an intron are alternatively spliced (AltB), and Exon Skipping (ES). The relative frequency of the various types of alternative splicing events is very similar to those observed in other plant species, with intron retention making up almost half of the events. Detailed statistics are provided in Table 1.

**Table 1:** The prevalence of different types of alternative splicing events.

	Chlamydomonas	Arabidopsis	Rice
IR	305 (50.0%)	4635 (56.1%)	7774 (53.5%)
ES	73 (11.9%)	666 (8.1%)	2004 (13.8%)
Alt5'	71 (11.6%)	845 (10.2%)	1642 (11.3%)
Alt3'	158 (25.8%)	1810 (26.0%)	2201 (15.5%)
AltB	4 (0.7%)	308 (3.7%)	921 (6.3%)

Total	611	8264	14542

This table shows the number and frequency of each type of alternative splicing. Percentage of the total number of events is shown in parentheses. The statistics for Arabidopsis and rice are from [11].

### Splice site strength

**Figure 2:**
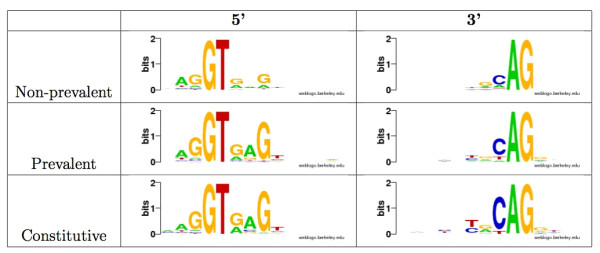
**Comparison of splice site motifs for prevalent and non-prevalent splice forms**. WebLogo [58] images of 5' and 3' splice site motifs for the prevalent and non-prevalent Alt5' and Alt3' splice forms. In the case of Alt5' there is a difference between the prevalent and non-prevalent forms only in the 5' splice site, and similarly for Alt3', there is a difference only in the 3' splice site.

We compared the splice site strength of the 5' and 3' splice sites in all types of alternative splicing events to those of constitutively spliced genes using the protocol described in [29]. Consistent with observations in other organisms [29], splice sites that participate in alternative splicing are weaker than constitutive splice sites, and all the differences are statisitically significant (see Table 2). The most significant difference is found at the 3' splice site of Alt3' events. In each alternative splicing event, we identified the prevalent splice form as the one supported by the largest number of ESTs. We observed that in the case of Alt5' and Alt3' events the splice sites for the non-prevalent splice forms are weaker than those of the prevalent splice form; the latter are weaker than those observed in constitutive splicing (see Table 3 and Figure 2). These differences are also highly statistically significant.

**Table 2:** Splice site strength in alternative and constitutive splicing.

Event	5' site		3' site	
	**motif score**	***p*-value**	**motif score**	***p*-value**

Intron Retention	7.790	7.492 (6.258e-44)	7.165	6.925 (8.734e-11)

Exon Skipping	6.701	6.156 (1.465e-09)	7.735	6.921 (4.402e-12)

Alt 5'	7.304	6.373 (7.175e-20)	7.448	7.097 (0.0517)

Alt 3'	8.594	8.434 (0.00176)	5.460	3.478 (1.686e-80)

Constitutive	8.822	N/A	7.574	N/A

Average splice site scores and *p*-values for alternative splicing events and constitutive splicing are shown here. All scores are computed with respect to the splice site motif of the constitutive splice form, following the protocol used in [29]. In all cases, the scores for the alternatively spliced form are lower than for constitutive splicing. The *p*-values are based on a comparison of the scores for each type of alternative splicing event with the scores for constitutive splicing, and are computed using the Wilcoxon signed-ranks test. Except for the case of exon skipping, the 5' and 3' sites refer to the splice sites of an excised intron. In exon skipping the 5' and 3' sites are the splice sites flanking the skipped exon.

**Table 3:** Splice site strength for prevalent and non-prevalent splice forms.

AS event	non-prevalent vs prevalent			prevalent vs constitutive		
	**non-prevalent avg. score**	***p*-value**	**prevalent avg. score**	**prevalent avg. score**	***p*-value**	**constitutive avg. score**

Alt5'	5.88	1.98e-07	8.14	7.51	1.67e-07	8.85

Alt3'	4.17	2.30e-12	6.73	6.31	6.95e-18	7.57

Splice site scores and *p*-values for the 5' and 3' sites of prevalent and non-prevalent splice forms. The table shows data that support two hypotheses: (i) non-prevalent splice sites are weaker than splice sites associated with the prevalent splice form; (ii) prevalent splice sites are weaker than splice sites associated with constitutive splicing. The "avg. score" columns provide the average score of splice site occurrences. For the comparison of non-prevalent with prevalent splice forms, the scores are computed with respect to a motif model of prevalent instances; for the comparison of prevalent and constitutive splicing the scores are computed with respect to a model of the constitutive splice sites.

### Length and GC content of retained introns and skipped exons

Alternatively spliced introns and exons in multicellular organisms were shown to have different length and nucleotide composition than their constitutively spliced counterparts [11,30]. We compared the length of retained introns and skipped exons with those that did not exhibit alternative splicing. This analysis revealed that retained introns are shorter than those that did not exhibit alternative splicing. The median size of retained introns is 127 bp compared to a median size of 232 bp in constitutively spliced introns. The difference is more pronounced in *Chlamydomonas *than in *Arabidopsis*, where median sizes are 93 bp compared to 100 bp [30]. Skipped exons are shorter than exons that are not known to exhibit alternative splicing with a median size of 84 bp compared to a median size of 132 bp in constitutively spliced exons.

In land plants, introns have high AT content, whereas exons are GC rich. Subsequently, a high percentage of A/T or T was reported to be important for efficient splicing of introns in flowering plants. Proteins that bind to U-rich stretches in pre-mRNA have been reported in plants [26,31]. In *Chlamydomonas*, we found that retained introns have a GC content of 57% as compared to 62% for constitutive introns. Furthermore, short, in-frame introns have an even lower GC content (56%). Similarly, skipped exons have a lower GC content as compared to constitutive exons (63% versus 66%). All these differences are highly statistically significant (*t*-tests yielded *p *values of 4.1475e-35, 2.5601e-06, and 0.0031, respectively). In Arabidopsis, the opposite trend is observed; retained introns have a higher GC content [30].

### Impact of alternative splicing on predicted proteins

Alternative splicing often results in the occurrence of a premature termination codon (PTC) [1,6]. Transcripts with PTCs are potential targets for degradation through non-sense mediated mRNA decay (NMD) [32,33]. Several recent studies suggest that the alternative splicing of pre-mRNAs is coupled to mRNA degradation through regulated unproductive splicing and translation, (RUST) [6,7,34]. To analyze predicted proteins, we focused on clusters that have full-length cDNAs and a single alternative splicing event so that we can predict the effect of alternative splicing on the resulting protein. Out of the 498 clusters showing AS, 483 correspond to annotated genes. Of these, 416 have published start codons, and 77 had a single AS event, and include a stop codon within a full-length EST. In 76 out of these 77 clusters, whenever alternative splicing occurred in the coding region, the non-prevalent splice form led to a shorter protein because of a PTC, resulting in a protein that is around 50% shorter (see Table 4). In Arabidopsis 78% of alternative splicing events occur in the coding region and about 50% of these have a PTC [11]. It has been shown in plants that transcripts with a PTC undergo NMD, and some of the components involved in NMD have been reported in plants [34-37]. Interestingly, the predicted proteome of *Chlamydomonas *contains components of NMD such as UPF3 and exon-junction complex proteins, suggesting that the NMD might play a role in the regulation of gene expression.

**Table 4:** The effect of splicing on predicted proteins.

	AS in Coding Sequence				
			
		ORF Shortened By			
				
Event	# events	bp	%	AS in UTR # events	Total
IR	30	276.52	54.52%	1	31
ES	4	270.00	59.74%	0	4
Alt5'	6	353.62	60.67%	4	11
Alt3'	22	476.32	51.37%	9	31

Total/Avg.	62	359.52	54.84%	14	77

We considered a subset of the clusters with a full-length cDNA, a single alternative splicing event, and a published start codon in the JGI version 4.0 genome annotation. For these clusters we show the number of events in a UTR and the number of events in the coding sequence, where UTRs were detected by the location of where AS occurred with respect to the published start codon and the first stop codon in the reading frame. For events in the coding sequence, we show the average reduction in the length of the predicted ORF that results when comparing the prevalent splice form with the non-prevalent splice form. In all cases but one, the non-prevalent splice form is shorter as a result of a premature termination codon. For IR, the prevalent form is always the one where the intron is spliced, and the non-prevalent form retains the intron. For ES, the prevalent form always contained the exon while the non-prevalent form skipped it.

### Alternative splicing motifs

Since introns have four loosely conserved signals, it is thought that other sequences may be involved in regulated splicing. In metazoans and land plants, protein factors such as SR proteins and hnRNPs have been shown to regulate splicing by binding to such splicing regulatory elements either in exons or introns and to enhance or prevent the usage of a splice site [38,39]. We performed a motif analysis of retained introns in comparison to constitutive introns using the DME program [40] (see details in the Additional file 1). While we didn't find statistically significant motifs in retained introns, constitutively spliced introns consistently produced such motifs. All the motifs were tandem repeats of di-nucleotides or tri-nucleotides. The consensus sequence for the top scoring motif was TGCTGCTG. A complete list of motifs with their associated p-values is presented in Table S4 in Additional file 1. Simple repetitive elements have been shown to bind splicing regulatory proteins such as SRs and hnRNPs and contribute to regulated splicing [41]. In *Chlamydomonas *there are several SR and hnRNP proteins that share significant sequence similarity with splicing regulators in multicellular organisms.

### Experimental verification of alternative splicing

**Figure 3:**
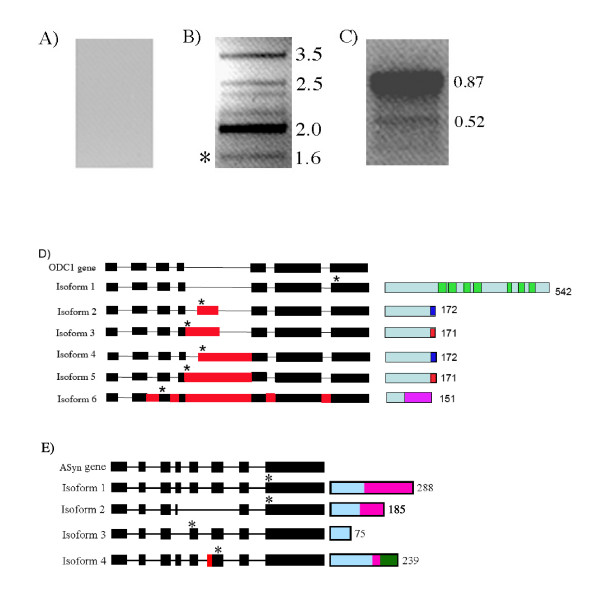
**Analysis of ornithine decarboxylase 1 (ODC1) and asparagine synthase (ASyn) splice variants in Chlamydomonas using RT-PCR**. A) DNAase-treated RNA was used in PCR with ODC1 primers. B) cDNA amplified with ODC1 primers. An asterisk indicates the spliced form for the full-length protein. Numbers on the right indicate amplified product size in Kb. C) cDNA amplified with ASyn primers. Numbers on the right indicate amplified product size in Kb. D) Diagram showing splicing events in six splice variants (left) and predicted proteins (right) for ODC1. Gene is indicated on top and all six splice variants are shown under the gene. Black boxes indicate constitutively spliced exons and red boxes indicate the included regions in different isoforms. Asterisk indicates the position of translation termination codon. Isoforms 1 to 6 correspond to the bottom to top bands in 3B. The number next to each predicted protein indicates the length of the protein. Conserved signature motifs in ODC1 are represented as green boxes in the full-length protein. Red, blue and magenta colors in truncated proteins represent amino acids unique to them. E) Diagram showing splicing events in four splice variants (left) and predicted proteins (right) for the gene ASyn. The representation of the gene and its splice variants is the same as in D. A conserved domain in ASyn is shown in pink. The green region in isoform 4 represents a unique sequence.

For experimental verification, we chose two of the clusters corresponding to ornithine decarboxylase 1 (ODC1, gene ID: OVA2_SAN_estEXT_fgenesh2_kg.C_340012) and asparagine synthase (ASyn, gene ID: estExt_fgenesh2_kg.C_280076), and performed reverse transcription PCR (RT-PCR). Amplification of DNAse-treated RNA with primers corresponding to these genes did not yield any products (Figure 3A), suggesting no DNA contamination in our RNA. Our RT-PCR analysis with primers corresponding to the first and last coding exons showed six splice variants with ODC1 (Figure 3B) and two splice variants with ASyn (Figure 3C). Alignments of previously-available ESTs predicted two alternative splicing events in ODC1 and three in Asyn. To identify the type of splicing events in each of these splice variants, we have cloned and sequenced all amplified products. The types of alternative splicing events and their influence on the predicted proteins are presented in Figures 3D and 3E. Complete nucleotide and predicted amino acid sequence of all splice variants for both genes are provided in Supplement 2. Our RT-PCR results show that ODC1 produces more isoforms than predicted from EST alignments, suggesting that the available ESTs/cDNAs because of their limited number, do not predict all alternative splicing events in a gene.

Sequence analysis has revealed that five of the six forms are due to alternative splicing of the 4th intron, which is the largest in this gene. In a study of alternative splicing of SR genes, which undergo extensive alternative splicing in Arabidopsis, it was found that in almost all SR genes the longest intron was involved in generating multiple transcripts by alternative splicing. The alternative splicing events observed in ODC1 include intron retention, Alt5' and Alt3' events. Only one of the six isoforms produced the full-length protein of 542 amino acids, which contains all of the seven conserved signature motifs of ODC1; the remaining five splice variants are predicted to produce three different truncated proteins with 151 to 172 amino acids due to in-frame translation termination codons. None of these three proteins contain conserved regions found in ODC1, hence are not likely to be functional. In humans, splice variants that have a premature termination codon at more than 50 nucleotides upstream of the last 3' exon-exon junction are known to be degraded by NMD [33]. Five of the six splice variants of ODC1 meet this criterion, hence are likely to be the targets of the NMD surveillance system. Interestingly, all five splice variants with a PTC are abundant and in some cases are present in higher levels when compared to the functional transcript (Fig. 3C, compare the lower band to the rest of the bands), suggesting some type of regulatory role for these other transcripts. Three of the four splice variants of the ASyn gene also encode truncated proteins (Figure 3E, Additional File 2). Two of these isoforms (isoform 3 and 4) are also likely targets of NMD. Of the three predicted splice variants for the ASyn gene we verified one of them (isoform 1), and detected a novel splice variant (isoform 2). ASyn transfers the amide group of glutamine to aspartate to form asparagine and plays a role in nitrogen metabolism [42]. Although land plants have one or more ASyn genes it is not known if the pre-mRNAs from these undergo alternative splicing [43].

Ornithine decarboxylase is a key rate-limiting enzyme in the biosynthesis of polyamines, which are required for cell growth and cell division in *Chlamydomonas *and other organisms [44]. It catalyzes the formation of putrescine from ornithine. ODC is present in algae and animals. However, higher plants such as Arabidopsis do not have ODC and synthesize polyamines via a different pathway [45]. ODC pre-mRNA in animals also undergoes alternative splicing. However, the 5' untranslated region is alternatively spliced in animals and this event controls ribosomal entry on the ODC mRNA [46]. The physiological significance of ODC1 isoforms in *Chlamydomonas *remains to be studied. It is possible that the PTC forms may be involved in regulating the level of the functional isoform through regulated unproductive translation and splicing. In addition to these two genes (ODC1 and ASyn) splice variant predictions for a few other genes were verified by others [47-52]. A recent analysis of thylakoid membrane proteins provides support for alternative splicing resulting in the generation of two proteins from a single gene [53].

## Conclusions

During the last six years, the estimates of the extent of alternative splicing in flowering plants has increased from 5% to 30% [3] due to an increase in available EST and full-length cDNA sequences. It is likely that the percentage of *Chlamydomonas *genes known to undergo alternative splicing will increase as more ESTs/cDNAs become available. Deep sequencing of the *Chlamydomonas *trascriptome under various conditions using next generation sequencing technologies should provide information on the real extent of alternative splicing. However, it is also likely that the prevalence of alternative splicing is roughly four-fold less than in flowering plants. We found that 3% of *Chlamydomonas *genes are alternatively spliced; in Arabidopsis, with a similar number of ESTs/cDNAs used in this analysis, about 12% of genes were predicted to be alternatively spliced [54].

There are only a few cases of alternative splicing reported in *Chlamydomonas *[47-52]. In several of these the protein coded by splice variants was found to be different, suggesting that proteins generated by alternative splicing may have different functions. In support of this, alternative splicing in some of these genes has been shown to have a physiological role. Hence, the observed alternative splicing events reported here are likely to be important in regulating gene expression and protein function. Furthermore, alternative splicing may also contribute to the regulation of functional transcript levels through RUST.

Our analysis indicates that alternative splicing is prevalent in *Chlamydomonas reinhardtii*. However, the extent of alternative splicing is much lower than what is observed in land plants. The frequency of different alternative splicing events is similar to flowering plants, with about half of all splicing events representing intron retention. Our finding that a large number of genes in *Chlamydomonas *undergo alternative splicing, together with the simplicity of the system and the availability of powerful experimental tools (molecular and genetic) suggest that this organism can serve as an attractive experimental system to understand the mechanisms involved in regulated splicing.

## Methods

### Detection of alternative splicing

To detect potential alternative splicing events, we obtained 252,484 high-fidelity Chlamydomonas EST sequences that Liang et al. corrected using cDNA termini to anchor transcripts to their correct positions in the genome [20]. The ESTs were aligned to the *Chlamydomonas *genome using the BLAT program [23]. We performed several filtering steps to obtain high quality alignments. The resulting alignments were clustered into putative transcriptional units and processed using a modified version of the Sircah tool to detect alternative splicing events. Details of our alignment processing and alternative splicing detection are found in Additional file 1.

### Chlamydomonas Culture

In our experiments we used two strains of *Chlamydomonas reinhardtii *(the wall-less strain (cc503) and wt (cc1690)). Both were obtained from the Chlamydomonas Center culture collection at Duke University. These strains were then grown in TAP medium [55]. The cultures were maintained at 22°C on a shaking platform in a growth chamber set on a 12:12 light/dark cycle [55]. Cells were subcultured during log phase at a starting density (determined by a hemocytometer) of 10^5 ^cells/mL [55]. For RNA isolation, a 2 ml aliquot was collected during log phase in 2 ml tubes and centrifuged at 0.2 g for 2 minutes. Supernatant was removed and the procedure repeated until cells from 4 to 6 mls were harvested in the same tube. The resulting pellet was then frozen immediately in liquid N_2 _and stored at -20°C.

### RNA Isolation and cDNA synthesis

Total RNA was isolated using an RNeasy Plant Mini Kit (Qiagen, http://www.qiagen.com/. Prior to RNA isolation, the cell pellet was thawed on ice and frozen in liquid N_2_. This procedure was repeated 2-3 times in order to lyse the cells and the total RNA was isolated according to the protocol provided by the kit manufacturer. RNA amount was quantified spectrophotometrically at 260 nm. The RNA sample was treated with DNase I according to the manufacturer's instructions (Invitrogen). The quality of RNA was verified by running an aliquot on a 1% agarose gel. DNase-treated RNA (1.5 *μg*) was used to synthesize first-strand cDNA with an oligo (dT) primer using SuperScriptII (Invitrogen).

### PCR of ODC1 and ASyn transcripts

One-twentieth of the first-strand cDNA was used for PCR amplification in a reaction volume of 20 *μ*l. The primers were designed using the Primer3 Input http://frodo.wi.mit.edu/ software. Touchdown PCR (TD-PCR) was performed using a temperature range of 50 - 60°C based upon the primer T_*m *_[56]. An extended hot-start method was utilized in which the PCR sample was allowed to incubate at 95°C for 1.5 hrs prior to PCR cycling. The following TD-PCR conditions were used: initial denaturation performed at 95°C for 3 minutes, followed by 10 cycles where denaturation was at 95°C for 30 seconds, and an annealing temperature of 60°C for 45 seconds. The annealing temperature was set to decrease 0.5°C every cycle until the 10 cycles were complete. Elongation was at 72°C for 3.5 minutes. The next 20 cycles had a denaturing temperature of 95°C for 30 seconds, an annealing temperature of 50°C for 45 seconds, and an elongation temperature of 72°C for 3.5 minutes. The final extension was at 72°C for 5 minutes. Amplified PCR products were resolved by electrophoresis in 1% agarose gels. All PCR reactions were performed using Takara EX Taq™ polymerase. Bands were extracted using a razor blade and stored at -20°C until gel extraction was performed.

### TOPO Cloning and Sequencing

Gel extraction was performed prior to TOPO cloning using the GeneJET™ Gel Extraction kit (Fermentas). After DNA was extracted, the sample was dried using the Speed-Vac and dissolved in 4 *μl *of water. The DNA was cloned using the TOPO TA Cloning Kit (Invitrogen). Plasmid from white colonies was isolated using the QIAprep Spin Miniprep kit (Qiagen). Inserts in plasmids were verified using PCR as well as digestion with EcoRI. Clones with an insert were then sequenced at Colorado State Macromolecular Center. Analysis of sequences was performed using the Spidey program [57].

## Authors' contributions

This study was conceived by AR, and designed by AR and AB. Bioinformatics analysis was carried out by AB, AL, and MR, and supervised by AB. Wetlab experiments were performed by ALink with the help of JT. AR and AB drafted the manuscript, with contributions from AL, MR, ALink, and JT. All authors read and approved the final manuscript.

## Supplementary Material

Additional file 1 **Supplementary Material**. The supplementary material contains additional tables and figures, and a more in-depth description of the alternative splicing detection and visualization pipeline. Click here for file

Additional file 2 **ODC1 and ASyn sequence information**. The file contains complete nucleotide and predicted amino acid sequences of all ODC1 and ASyn splice variants. Click here for file
