# Supplementary material for: Genome-wide analysis of alternative splicing in Chlamydomonas reinhardtii
Source: BMC Genomics. 2010 Feb 17;11:114. doi: 10.1186/1471-2164-11-114 (PMC2830987; doi:10.1186/1471-2164-11-114)
Supplement: Additional file 1 — Supplementary Material. The supplementary material contains additional tables and figures, and a more in-depth description of the alternative splicing detection and visualization pipeline. [file 1471-2164-11-114-S1.PDF]

# Supplementary material for "Genome-Wide Analysis of Alternative splicing in *Chlamydomonas reinhardtii*"

December 2, 2009

## 1 Some properties of the *Chlamydomonas* genome

Table 1 presents genome statistics for *Chalmydomonas* as compared to other organisms. Data in the table is compiled from several sources [6, 10], and the *Chlamydomonas* version 4.0 genome annotations.

| Organism | % of genes with introns | Avg. # Exons per gene | Avg. intron length | Avg. exon length | G+C % in genome |
|---|---|---|---|---|---|
| *Chlamydomonas* | 88 | 7.4 | 336 | 240 | 64 |
| *S. cerevisiae* | 5 | 1 | 256 | 1500 | 38 |
| *S. pombe* | 43 | 2 | 107 | 330 | 36 |
| Arabidopsis | 79 | 5 | 165 | 304 | 36 |
| Human | 85 | 9 | ∼3400 | 282 | 41 |

Table 1: A comparison of the *Chlamydomonas* genome.

## 2 Detection of alternative splicing from EST-to-genome alignments

In what follows we describe the steps in our pipeline for aligning and detecting alternative splicing. An overview of the process is shown in Figure 1.

### 2.1 EST alignment and clustering

To detect potential AS events, we obtained high-fidelity *Chlamydomonas* EST sequences that Liang et al. corrected using cDNA termini to anchor transcripts to their correct positions in the genome [7]. The 252,484 EST sequences were aligned to the *Chlamydomonas* v4.0 draft genome [4] with the `BLAT` [5] program. Default parameters were used except for maximum intron length, which was set to 2000 as determined by

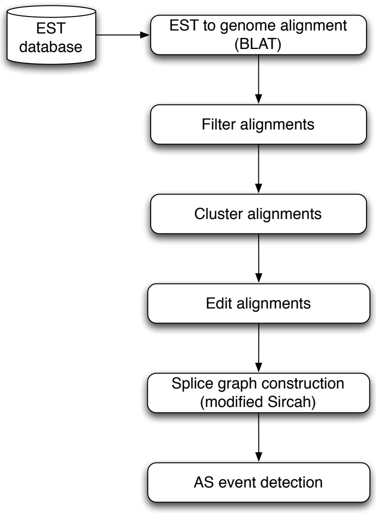


Figure 1: The steps in the alternative splicing detection pipeline. See text for details.

analysis of intron lengths in the genome. The resulting alignments were filtered such that the total number of matched bases was greater than or equal to 90% of the length of the EST. Out of all alignments for a given EST, the alignment with the highest percent identity was identified. These alignments were further filtered to include only alignments with all canonical GT/AG splice sites.

The final set of alignments underwent preprocessing to reduce sequence and alignment artifacts. Inserts in the EST sequence with respect to the genome were removed such that alignments had no genomic gaps. Short deletions in an EST were considered a result of polymorphism between *Chlamydomonas* strains, and were ignored. Finally, initial and terminal exons were filtered if they were shorter than 10 bases to avoid detecting potentially spurious AS events due to incorrect alignments. The edited alignments were grouped into clusters such that all EST alignments in a cluster have overlapping genomic coordinates and they align to the same strand.

From the alignments remaining after filtering we constructed 8177 clusters of overlapping ESTs. We successfully mapped 7675 out of the 8177 clusters to genes in v4.0 of the *Chlamydomonas* genome using overlapping positions in the genome to identify matches. We processed the EST clusters, and used a modified Sircah implementation (described below) to identify genes with evidence of AS.

## 2.2 Splice graph construction and prediction of AS events

We used Sircah [3] to detect AS events from the clusters of aligned ESTs. Sircah is an application written in Python that detects AS events and provides visualizations in the form of splice graphs. On the program's website [1], the authors have outlined the rules Sircah uses to detect AS events. Those rules were developed in the context of metazoans where intron retention is rare. However, their rules may detect spurious alternative 3' and 5' events whenever there is intron retention: Sircah detects alternative 3' or 5'

splice sites whenever an exon in one transcript overlaps an exon with a distinct 3' or 5' splice site in another transcript (see Figure 2). We modified Sircah's rules to eliminate such spurious detection of Alt5' and Alt3' events.

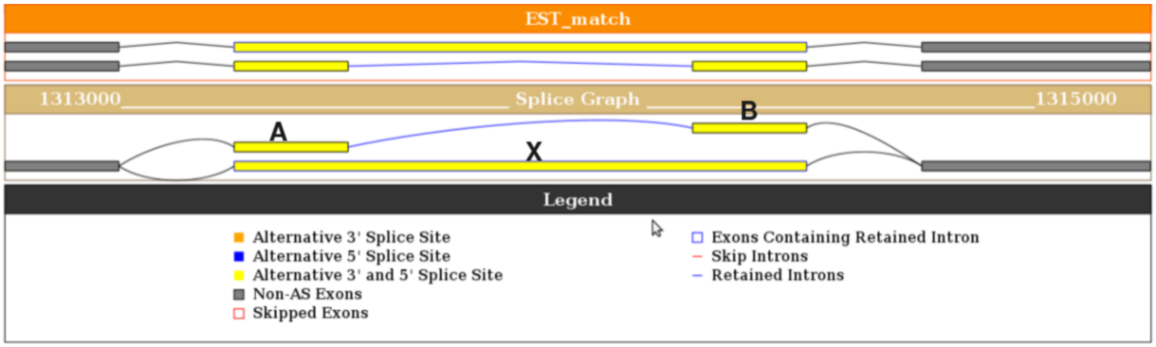


Figure 2: This splice graph shows how the original Sircah rules interpret an intron retention event. Sircah's algorithm may detect alternative 3' and 5' exons when evidence supports only an intron retention event. Here, Sircah detected that $X$ overlaps $A$ and that their 3' splice sites are distinct. Similarly, it detected that $X$ overlaps $B$ and that their 5' splice sites are distinct. As a result, it highlighted spurious alternative 5' and 3' events.

# 3 Properties of alternative splicing events

Using the modified Sircah we identified AS events and generated a variety of statistics for each kind of AS event.

**Retained introns.** We compared the intron length distributions for constitutive introns and retained introns (Figure 3); we observe that retained introns tend to be shorter (median: 127 bp compared to a median of 232 bp in constitutively spliced introns). In addition, we found a statistically significant difference in the GC content of retained introns compared to constitutive introns (57.8% vs. 63.7%, $p < 10^{-21}$). See also Table 2. We also checked whether in-frame intron retention events (retained introns whose length is divisible by 3) were over-represented in our data. This would be the case if significantly more than a third of IR events were in-frame, but out of 325 IR events, 108—almost exactly one-third—were in-frame.

**Alt5' and Alt3' events.** For Alt5' and Alt3' events we examined the change in exon length as a result of each such event. The distribution of these offsets is shown in Figure 4.

## 3.1 The impact of alternative splicing on predicted proteins

To find the effect each kind of AS event has on a gene's product we compared the predicted protein produced by a gene's most common splice form (prevalent) with

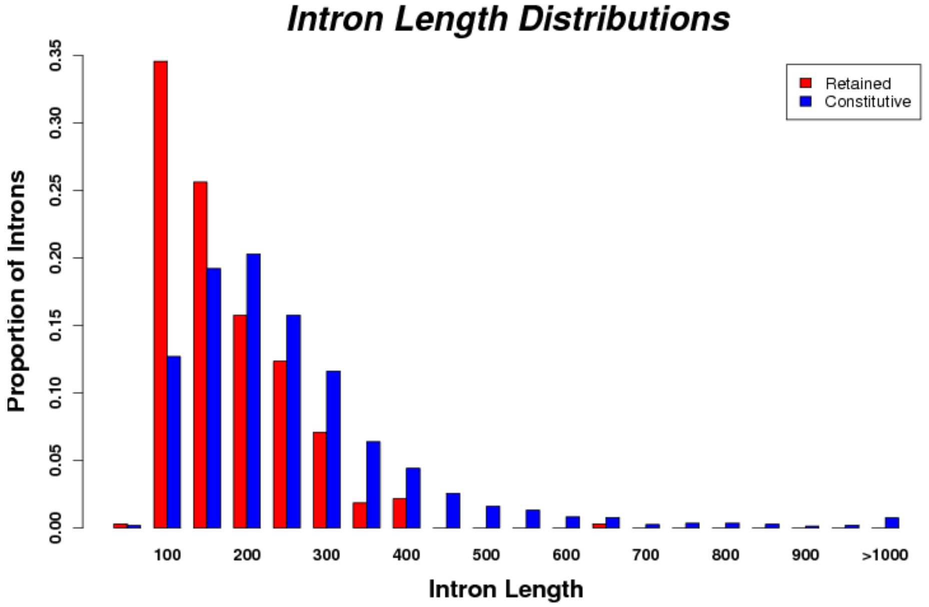


Figure 3: Comparison of intron length distributions between constitutively spliced introns and those involved in intron retention. Retained introns tend to be shorter than constitutive introns (median: 127 bp compared to a median of 232 bp in constitutively spliced introns).

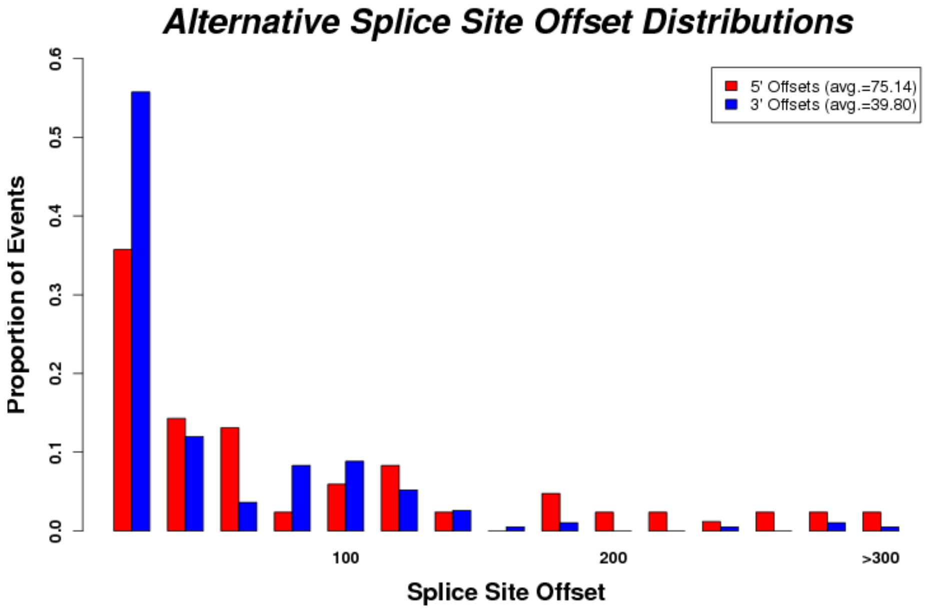


Figure 4: Distribution of the change in intron length across all alternative 3' and 5' splice site events. The majority of offsets are less than 40nt, with nearly 9% being 4nt long.

| | Introns | Exons |
|---|---|---|
| Single-exon | - | 63.0% |
| Non-AS | 62.4% | 66.4% |
| ES | 61.3% | 63.4% |
| IR | 57.8% | 60.4% |

Table 2: GC content for unspliced, constitutitvely spliced, and alternatively spliced genes. For intron retention we report the GC content for the intron and for flanking exons. For exon skipping we report GC content for the exon and for its flanking introns.

| AS event | 5' UTR | 3' UTR | ORF | Total |
|---|---|---|---|---|
| IR exon | 24 | 11 | 219 | 254 |
| ES exon | 5 | 3 | 69 | 77 |
| Alt. 3' site | 5 | 12 | 138 | 155 |
| Alt. 5' site | 8 | 1 | 50 | 69 |
| Total | 42 | 27 | 476 | 545 |

Table 3: Distribution of AS events within the 5' UTR, 3' UTR and the coding region for the clusters that had published start codons. Out of 447 clusters with published start codons, 64 had AS events within UTRs. In all, 13% of AS events occurred in UTR, while 87% occurred within open reading frames.

those produced in its alternative (non-prevalent) forms. We defined the *prevalent* form of a gene exhibiting alternative splicing as the splice form supported by the greatest number of ESTs. When possible, we used the longest transcript corresponding to the cluster's prevalent form as a reference. In cases where there were no ESTs spanning the full splice graph, we used the splice graph to construct the prevalent form and its alternative. For simplicity, and to isolate the effect of each AS event, we only performed this analysis on clusters with a single AS event.

We identified introns and exons in our splice graphs by their aligned positions in the reference genome. To convert their genomic sequences into putative proteins, we established an ORF using codon information from the JGI gene annotations. In some cases the start codon associated with a gene was far upstream of the first exon in our cluster. We omitted those clusters from our analysis. 82 clusters satisifed these criteria. Despite this relatively small sample, we found strong evidence that non-prevalent AS events yield truncated protein sequences as a result of a premature termination codon (results shown in the main paper).

To better understand how often AS events take place in the 3' or 5' UTR, we also used the published codon information to count the number of AS events in our clusters that appeared outside the ORF. We counted events upstream of a start codon as 5' UTR events; those downstream of a stop codon were 3' UTR events. Overall, 12.6% of AS events occurred in either the 3' UTR or 5' UTR, while 87.4% occured within the reading frame (see Table 3).

## 3.2 Splice site motif analysis

Sequences corresponding to AS events identified by the pipeline were analyzed for splice site strength following the protocol in [11]. Splice sites for each type of AS event (5', 3', intron retention, exon skipping) as well as splice sites where we found no evidence of AS were used to construct motifs using the TAMO package [2]. Splice sites were considered to be 3 bases of exonic sequence and 10 bases of intronic sequence flanking appropriate GT/AG splice site markers, resulting in motifs of length 13. Splice site instances were scored according to:

$$score = \sum_{j=0}^{N} \log \left( \frac{p_m(s(j), j)}{p_{bg}(s(j), j)} \right), \tag{1}$$

where $N$ is the length of the motif, $s(j)$ is the nucleotide at position $j$, $p_m(i, j)$ is the probability of seeing nucleotide $i$ at position $j$ of the motif, and $p_{bg}(i, j)$ is the background distribution for nucleotide $i$ at position $j$ of the motif. For the background distribution we used a background based on exon sequences for the exonic part of the motif, and a background based on intronic sequences for the intronic part of the motif. Two sets of motif instances were scored using Eqn. (1), and the significance of the difference between the scores was determined using the Wilcoxon Rank Sum test. $z$-scores were calculated using the normal approximation of the test and converted to $p$-values. In the paper we show comparisons between splice sites associated with AS events and constitutive splicing, showing that AS-associated splice sites are significantly weaker than constitutive ones; we also show that splice sites associated with non-prevalent splice forms are weaker than those of the prevalent splice forms, and that the splice sites of the prevalent splice forms are weaker than those of constitutive splicing.

## 3.3 Motif analysis

The objective of our motif analysis was to look for motifs that are over-represented in retained introns compared to constitutive introns (potential intronic splicing suppressors), and for motifs that are over-represented in constitutive introns compared to retained introns (potential intronic splicing enhancers). In this analysis we used the Discriminative Matrix Enumerator (DME) program [9]. DME is a discriminative motif finder, i.e., it takes as input a set of sequences of interest, and a set of background sequences where we don't expect to find the motifs. DME scores a motif occurrence according to the log odds ratio of its occurrence in the set of sequences of interest relative to the background set.

When using such a method the choice of the background is important: if the sequences of interest have completely different characteristics, then the significance of the discovered motifs can be exaggerated. In our case the two sets of sequences have different GC content and different length (DME does not normalize its score for length). The authors of DME recommend searching for motifs using more than one choice of background. We considered two types of background sets.

| Type of AS | 5’ Splice Site | 3’ Splice Site |
|---|---|---|
| Intron Retention | 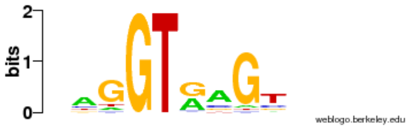 | 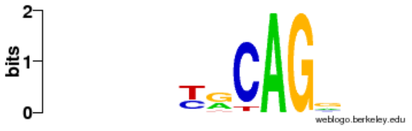 |
| Exon Skipping | 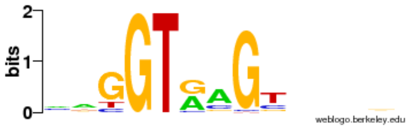 | 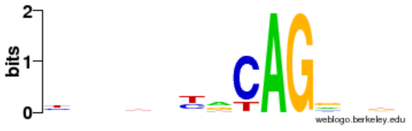 |
| Alt5’ | 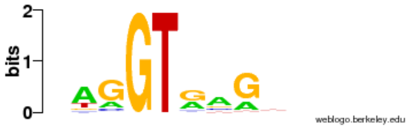 | 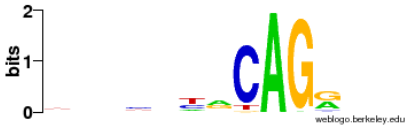 |
| Alt3’ | 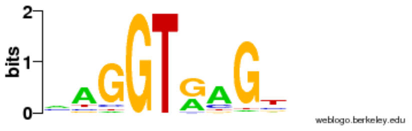 | 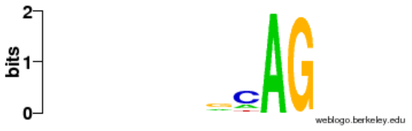 |
| constitutive | 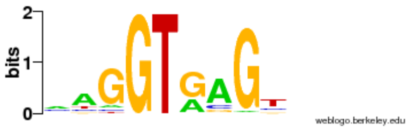 | 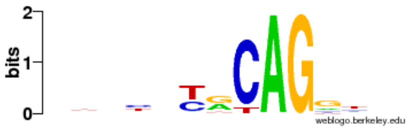 |

Figure 5: WebLogo images of splice site motifs for different types of AS. Except for the case of exon skipping, the 5’ and 3’ sites refer to the splice sites of an excised intron. In exon skipping the 5’ and 3’ sites are the splice sites flanking the skipped exon.

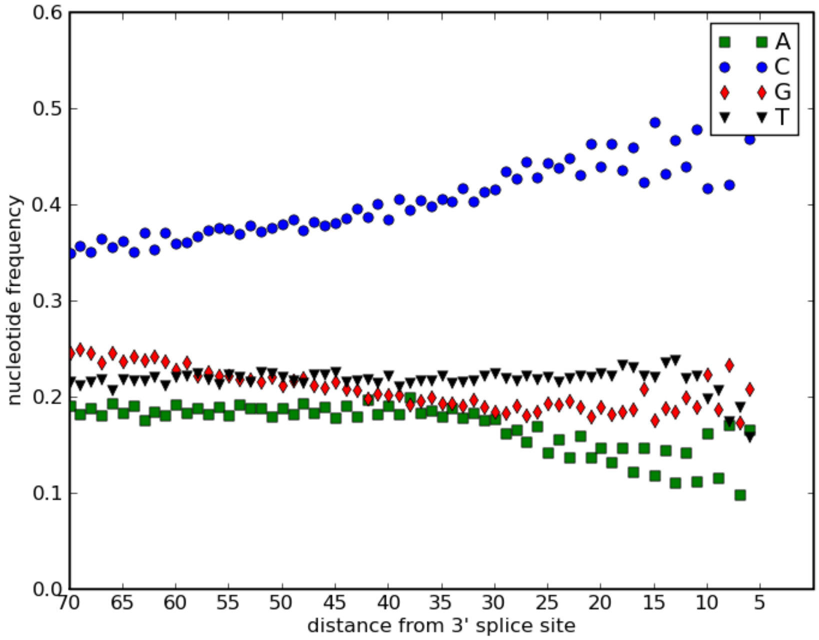


Figure 6: Nucleotide composition in the last 70bp of *Chlamydomonas* introns.

**Markov background.** Constructed to have the same di-nucleotide frequency as the foreground set. Sequence length is chosen as the average sequence length of the foreground set.

**Edited background.** Another way to account for the difference between the foreground and background sequences is to modify the background to have similar characteristics as the foreground. When using constitutively spliced introns as foreground, we "padded" the background (retained introns) with 4-mers from the foreground, which also made the GC content similar. When using retained introns as foreground, we randomly removed nucleotides from the background set to achieve similar length; to achieve similar GC content we biased the removal process appropriately.

**Assessing significance.** We assessed the significance of the discovered motifs by computing an empirical p-value. This was done by pooling the background and foreground sets and randomly dividing them into foreground and background sets, and running DME on the permuted background-foreground sets of sequences. The p-value of a motif was computed as the fraction of runs in which we found a motif with a score equal or higher to the score of the given motif.

**Results.** We ran DME looking for motifs of width 8 using its default parameters. Our search for motifs in retained introns yielded significant motifs when using the Markov

| Background | Consensus | p-value |
|---|---|---|
| Markov | TGCTGCTG | 0.0 |
| | CTGCTGCT | 0.001 |
| | TGTGTGTG | 0.002 |
| | CACACGCA | 0.015 |
| | GTGTATGT | 0.054 |
| | GTGCGTGT | 0.081 |
| edited retained introns | CTGCTGCT | 0.001 |
| | CAGCAGCA | 0.002 |
| | ACCCCCAC | 0.024 |
| | GCACACAC | 0.027 |
| | CGCACACA | 0.032 |
| | GTATGTGT | 0.073 |

Table 4: The top scoring motifs found in constitutively spliced introns with two kinds of background sequences. The Markov background is generated by a one-step Markov model with a transition matrix trained on the constitutively spliced introns; the "edited retained introns" sequences are obtained by randomly inserting 4-mers from the constitutive introns to obtain a similar length distribution. The p-value is an empirical p-value. See text for details.

background, but not when using the "edited" background. The run on a sample of constitutive introns gave significant motifs under both kinds of background, and the top scoring motif under both kinds of background was almost identical—a tandem repeat of *TGC*. Table 4 lists the top scoring motifs discovered using both kinds of background sets.

Comment: Since version 2 of DME searches for motifs on both strands, we had to modify the program so that it would only search on the positive strand.

# References


[1] Bork Lab. Sircah: Description. `http://www.bork.embl.de/Sircah/description.html`, May 2009.

[2] D. Benjamin Gordon, Lena Nekludova, Scott McCallum, and Ernest Fraenkel. Tamo: a flexible, object-oriented framework for analyzing transcriptional regulation using dna-sequence motifs. *Bioinformatics*, 21(14):3164–3165, 2005.

[3] E.D. Harrington and P. Bork. Sircah: a tool for the detection and visualization of alternative transcripts. *Bioinformatics*, 24(17):1959, 2008.

[4] Joint Genome Institute. Chlamydomonas reinhardtii v4.0. `http://genome.jgi-psf.org/Chlre4/Chlre4.download.ftp.html`, May 2009.

[5] W. James Kent. Blat - the blast-like alignment tool. *Genome Research*, 12(4):656–664, 2002.

[6] D.M. Kupfer, S.D. Drabenstot, K.L. Buchanan, H. Lai, H. Zhu, D.W. Dyer, B.A. Roe, and J.W. Murphy. Introns and Splicing Elements of Five Diverse Fungi Supplemental material for this article may be found at http://ec. asm. org/. *Eukaryotic Cell*, 3(5):1088–1100, 2004.

[7] C. Liang, Y. Liu, L. Liu, A.C. Davis, Y. Shen, and Q.Q. Li. Expressed sequence tags with cDNA termini: previously overlooked resources for gene annotation and transcriptome exploration in Chlamydomonas reinhardtii. *Genetics*, 179(1):83, 2008.

[8] S.S. Merchant, S.E. Prochnik, O. Vallon, E.H. Harris, S.J. Karpowicz, G.B. Witman, A. Terry, A. Salamov, L.K. Fritz-Laylin, L. Marechal-Drouard, et al. The Chlamydomonas genome reveals the evolution of key animal and plant functions. *Science*, 318(5848):245, 2007.

[9] A.D. Smith, P. Sumazin, and M.Q. Zhang. Identifying tissue-selective transcription factor binding sites in vertebrate promoters. *Proceedings of the National Academy of Sciences*, 102(5):1560–1565, 2005.

[10] V. Wood, R. Gwilliam, M.A. Rajandream, M. Lyne, R. Lyne, A. Stewart, J. Sgouros, N. Peat, J. Hayles, S. Baker, et al. The genome sequence of Schizosaccharomyces pombe. *Nature*, 415(6874):871–880, 2002.

[11] Christina L. Zheng, Xiang-Dong Fu, and Michael Gribskov. Characteristics and regulatory elements defining constitutive splicing and different modes of alternative splicing in human and mouse. *RNA*, 11(12):1777–1787, 2005.
