# Supplementary material for: Genome-wide analysis of alternative splicing in Chlamydomonas reinhardtii
Source: BMC Genomics. 2010 Feb 17;11:114. doi: 10.1186/1471-2164-11-114 (PMC2830987; doi:10.1186/1471-2164-11-114)
Supplement: Additional file 2 — ODC1 and ASyn sequence information. The file contains complete nucleotide and predicted amino acid sequences of all ODC1 and ASyn splice variants. [file 1471-2164-11-114-S2.DOC]

**A) ODC1 splice variants**

**Genomic Clone (4412 bases)**

GCCTGCCTGACCTTGCAGCCACCTCGGCTTCGGTACTCTCGCTTTACTTC

CCAGCTCGAGCTATCGCTCTCGCCTAGCGTTCACGCTGGCGCCCCGCAAT

CTCGCAACCGCTCTTTTAGCGCGCAGCTCTACTATCCTGTGCAGCCTAGG

CTCTCAGGCTATCATCCCAAGGTCTACTCCAGTCGTTC GGTGAGTTCCGG

GCTCCTTCCCTTGCCTCCGAGGCGCGGCGGCTGCGCGCGTCTTCCGCTGG

GTTCGTGTTGGCTGCAATGGCGGCTTGCTGCGCATCTAGGGCCATTTTTC

GTTTCAAGACGGCGCAGTCGGGGCACAGTCGTCACCTGTCGGCGACCGCG

CGGCATTCGCTCGCTGTCCACTTTATGCGACCGACTGGTTGACCTTGAGC

TTCTTCGCATCCTTTCCGCAGGTCCAACCGCGTCATCATGACAACTTCCG

AGGTTCTGAGTCC CCCGCCCCCGAGCCAGGTGGCTAGCCTGGCTCAGCCG

CAGTTCAACGTGGAGGCGCTTCAGGCCTTCTCGATGCCCCTGGTGGCCGA

CGTTGTCAACACTGAAAATGGCGACGTCGCTTCGTTCGCCCGCAGCTCCT

GGCACAAGGAGTCGGGCAAGGCGGGGGCGGTGAGTTTTCAGAGTTTGACG

ATCGCGGAATTCCCCGGTTGTCGGCTGCCTTCGGCCTTGGGCCAGCGCCC

CGCGCGCTTGTACTTGTGTGGCACACATTTAGTGGTTTGGGGGACCGGAT

CCGGTGATCGGCCGAGCGTCTGTCACCAGCCCATCAGGCCCTCGTGGCCT

TTGCTAAGCGCCGCTCGCGATCTGGCCTCATTTCCTAACCCAATTCTCTC

TGCATGTGCGCGCAGTTCGGCCTGCCGACCATGCTGGATATGAAGGCTGA

GGCTCTGGAGCAGGTGCAGCTGGACACCTGGTCGGCGCAGCCTGTGGACC

GTGGTCCGGCAGCAGCGGCTCCTGACCCCAAGCTGCTGCGCTCCTGGCAG

TACCAGGCTACTGGCAAGCTTGCAACCGCTCAGGTCCTGGTGGACGTCAA

GGCTGAGCACATCAAGGCTGGTGAGTTCCTAGGCTCACGTGGGACTGGGG

GCAGACCGTAGACGCAGACTGGCTAAGGAGTCTGGCTGCCGTTCCAGGCG

AAACGAGGTGCATGTGGGTTTGCCGGACCCCGTTGTGAGTGTGTGATCAG

TGAAGTCAGCCATCTACAAATTTGTAAGATTGTCGACTGATTATTGCTGT

TTTTTCTCTTCCCACGTAGGTGGCCCCATGGGCATGAAGCAGGAGGCCAT

TAAGCACATCCTGCGCCACAAGCCGGACGACACCTTCTATGTGGTTGACC

TGGCTAACGTGCAGCGCATGTTCAAG GTAATGAATTGATCCTCGCCGTCG

CGACTACCAGCGCGCGCGAGCGGGGTGGGTCGGGACCATGGTTGGGTTTC

GTTCATGCCTGTGGGTTGGGCTGCGCCGTGCATGCCATGGCGCCTGGTTG

CGTAGCGGAGCGCGCGAGAGCGAGCGGCACGGCGAATGAGCATGCGCAAT

TCCGAATTAACTCGGCCCTGGTGTGCTGTCCTATGTTCGACCTCGCAG GC

TAGCCCTGCCTGACTTTTCATTCGCAACCATTAGGCATGTGCCCCCACAC

ATCCCCACCACACCACCACCACCACGCGCCCGGCATCGCGTCATCTACAT

CCACGCCTGCATCAAGCTGCTCTGCTCGGCCTGGAGTCATCCGGGCCTTG

CCATCAGCGCCAAGTGCACGTCAACGCACACGCCTCACCAAGCGCAAGCC

TTCTCCTTCATGGGCAAGGTCTTAAGCGGTGGTGGTGGTGGTGGCGGCTG

GCACCTGGAGACGGAGCGTGGAGCGTGGAGCGGAGCCCGAAGACCCAGCA

TCGTTGCAAACAGCACAACCCCTGTTGCTCCTGGTAACCCTCTCGCATAC

TCACGCGCCATACTAATTGGGCCCGTTGTCTCTCTCTTGTCTTAGCAGCT

AGGTGTCTACTGGGCGCCATATGCACCGCATGAGTCTTTGTGCCTTGTGC

CGTTCGCGTGTGGCGTGTTGAGCATGCGCCTGCGTGTACGCGTGTTTGTC

TGTGCGGTTTTTTTGTGCCAGTCCGAGTGAGTCCTGGGCACGTGACCGCT

CACATGGCTCCTGGACCCAGCGCGCGTTAATCGCTGGCGCGCCCCCCTAT

GGGGCGCGTTGATGCTTCGCGTTGCATGGACTAGGTAGCATTGTGCCTCT

GTCTTGTCGTTGTCTCGCGTGTGCACACGCATGAAGCCACGTGGCGACCT

CCTGGTCTACAAAATCCATCAGACCAGAGAGCGCGCGCCATCACGGCCAG

GAGGGCCCGTGGCTACCTTCGGCGCCTTCTGATTTCCCAACTTGTCCCAT

CAAGTTGGGATGGAAGGAATTGCCAACTCCAGGTGGTTGCTGACGTTGTG

CGTTCCGTTTTCTCCTCTCCCTCAG GCCTGGCGCGCCGCTATGCCCCGCG

TGGTCCCCTTCTACGCGGTCAAGTGCAACCCGGAGCCGGGTATCCTGAAG

CTGCTGAACGCGCTGGGTGCTGGCTTCGACTGCGCTAGCAAGGGCGAGCT

GGACATGATGCTGCGCATGGGCGTCTCGCCCAACCGCATTATCTTTGCTC

ACCCCTGCAAGCGCGCCTCGGACATCCGCTACGCGCGCGAGCACAACGTG

CAGTACACCACGGTGAGTGTGCTGGGGCTGGGGAATGGGCAGTGGGACGG

CACTCGCTTCTGGGTCCAAGCATTGTATTTGTGGCAAGGAAAGCTCTTCA

CTGCAACGCTGACTTGTTACGTTGCCTTGCACCGTTTACCCGCAGTTCGA

CACCGTGTCGGAGCTGCACAAGATCGCGCAGATGAACCCCGACTTCAAGT

GCGTGCTGCGCATCCGCGCCGACGACCCGGACGCCCGCGTGCCTCTGGGC

CTGAAGTACGGCGCTGAGGTGTCGGAGGCCGACGTGCTGCTGCGCACGGC

GAAGGAGCTGGGCCTGCAGGTGGTGGGCGTCAGCTTCCACGTTGGCAGCG

CTTGCCAGAACCTGTCCACCTTCTCGGGCGCCATTGAGAACGCGCGCAAG

GTGTTCGACGAGGCCGGCGCCCTGGGCTTCAACATGGAGCTGCTGGATAT

CGGCGGTGGCTTCACCGGCCACTTCGACGAGATGGGCAACGTGATGTTCG

GCGAGATCGCGAACACCATCAACGCCGCTCTGGCCACCAACTTCCCGCCG

GAGATGGGCGTGCGCGTCATCGCCGAGCCCGGCCGCTACTTCGCTGAGAC

CAGCAGCACGCTGCTGACCCCCGTCTACGGCCAGCGCGACCGCGTGGCTG

CCGACGGCTCCGTGAAGAAGGACTACTGGCTCACCGACGGCCTGTACGGC

TCCTTCAACTGCATTCTGTACGACGGCCAGAACCCTGGCTACAAGGTCGT

GCGCTCGCCGCTGATGGCCGACTCGACCGACAGCCGCACCTTCCTGTCGA

CCCTGTGGGGTCCCACCTGCGACAGCGCTGACTGCGTCTACAAGGACGTG

ACCCTGCCCGTGCTGCGCAACGGTGACTGGCTGATGTGGAACAACGCCGG

CGCCTACACCGTGGCCGGTGCCTGCGACTTCAACGGCATCGAGTTCACCA

CTGTGAGTTTGCCATCAACCATCGGCTCTTTTGTTCGGTCGGGTGCACGG

TGTAAGGGTCATTTGATGATTTATGCTGCTATCATCTGTACCGATATCCA

ACTGACCTCCAACTTGTCCTTTTGCGCGCAGCCGGGCAAG CTGTACGTGT

GGTCCGACAGCGCGGTGGACGCGGCCGAGGAGGGCGCTGACGAGCAGGTC

ATGAACGCGTGAACGCAACGTGACAGCACTTCGCGATTGCTCTCGTGATA

TTTGCGCGCGGGATGACCCGCTCTGTCTGCATTTCTGTTTATTGGATGGT

TTGTTGTCCGCCCTTGAATGAGGGCCACGATGGGCACGGGGGCGTGGTGT

CAGATGTGGTCCGGCCACAGCTTGGGCGTGTGAAAGTCTCAGGTTGGGTT

TTGTTCATAGTCGCCGGGCACGCCTGACCGGCCCCGCTGCTAAAATTGCC

ACAGGACTAGCAAACTGCATTATGAAGTTTTTGGGACGGAATTTGTCACT

ATACTTGTATTTCTGGCCCCGCGAGATGCACGGGTGTCAGTCGAGGAGGG

GCAGACCGCCTCCGCCGGAGAAAGATGTACAGGGTACGCAGGGTGTATGC

AGGTTAGACATGCAGATGGACCGGTCGATTTTGGATGAGGAGGATTGCAG

CACGGACTGGATGCGATTGGACCTGGAACGTGTAACGAAAGAACGATGAT

GCATTGGTCCCGTCCAGTGGGGACGTGGATGCTGTCTAGTCAGGATTTTG

ACCATCAGGATT

**Isoform 1**

ATGACAACTTCCGAGGTTCTGAGTCCCCCGCCCCCGAGCCAGGTG

GCTAGCCTGGCTCAGCCGCAGTTCAACGTGGAGGCGCTTCAGGCCTTCTC

GATGCCCCTGGTGGCCGACGTTGTCAACACTGAAAATGGCGACGTCGCTT

CGTTCGCCCGCAGCTCCTGGCACAAGGAGTCGGGCAAGGCGGGGGCGTTC

GGCCTGCCGACCATGCTGGATATGAAGGCTGAGGCTCTGGAGCAGGTGCA

GCTGGACACCTGGTCGGCGCAGCCTGTGGACCGTGGTCCGGCAGCAGCGG

CTCCTGACCCCAAGCTGCTGCGCTCCTGGCAGTACCAGGCTACTGGCAAG

CTTGCAACCGCTCAGGTCCTGGTGGACGTCAAGGCTGAGCACATCAAGGC

TGGTGGCCCCATGGGCATGAAGCAGGAGGCCATTAAGCACATCCTGCGCC

ACAAGCCGGACGACACCTTCTATGTGGTTGACCTGGCTAACGTGCAGCGC

ATGTTCAAGGCCTGGCGCGCCGCTATGCCCCGCGTGGTCCCCTTCTACGC

GGTCAAGTGCAACCCGGAGCCGGGTATCCTGAAGCTGCTGAACGCGCTGG

GTGCTGGCTTCGACTGCGCTAGCAAGGGCGAGCTGGACATGATGCTGCGC

ATGGGCGTCTCGCCCAACCGCATTATCTTTGCTCACCCCTGCAAGCGCGC

CTCGGACATCCGCTACGCGCGCGAGCACAACGTGCAGTACACCACGTTCG

ACACCGTGTCGGAGCTGCACAAGATCGCGCAGATGAACCCCGACTTCAAG

TGCGTGCTGCGCATCCGCGCCGACGACCCGGACGCCCGCGTGCCTCTGGG

CCTGAAGTACGGCGCTGAGGTGTCGGAGGCCGACGTGCTGCTGCGCACGG

CGAAGGAGCTGGGCCTGCAGGTGGTGGGCGTCAGCTTCCACGTTGGCAGC

GCTTGCCAGAACCTGTCCACCTTCTCGGGCGCCATTGAGAACGCGCGCAA

GGTGTTCGACGAGGCCGGCGCCCTGGGCTTCAACATGGAGCTGCTGGATA

TCGGCGGTGGCTTCACCGGCCACTTCGACGAGATGGGCAACGTGATGTTC

GGCGAGATCGCGAACACCATCAACGCCGCTCTGGCCACCAACTTCCCGCC

GGAGATGGGCGTGCGCGTCATCGCCGAGCCCGGCCGCTACTTCGCTGAGA

CCAGCAGCACGCTGCTGACCCCCGTCTACGGCCAGCGCGACCGCGTGGCT

GCCGACGGCTCCGTGAAGAAGGACTACTGGCTCACCGACGGCCTGTACGG

CTCCTTCAACTGCATTCTGTACGACGGCCAGAACCCTGGCTACAAGGTCG

TGCGCTCGCCGCTGATGGCCGACTCGACCGACAGCCGCACCTTCCTGTCG

ACCCTGTGGGGTCCCACCTGCGACAGCGCTGACTGCGTCTACAAGGACGT

GACCCTGCCCGTGCTGCGCAACGGTGACTGGCTGATGTGGAACAACGCCG

GCGCCTACACCGTGGCCGGTGCCTGCGACTTCAACGGCATCGAGTTCACC

ACTCCGGGCAAGCTGTACGTGTGGTCCGACAGCGCGGTGGACGCGGCCGA

GGAGGGCGCTGACGAGCAGGTCATGAACGCGTGA

**Isoform 1 protein: (542 aa)**

MTTSEVLSPPPPSQVASLAQPQFNVEALQAFSMPLVADVVNTENGDVASFARSSWHKESGKAGAFGLPTMLDMKAEALEQVQLDTWSAQPVDRGPAAAAPDPKLLRSWQYQATGKLATAQVLVDVKAEHIKAGGPMGMKQEAIKHILRHKPDDTFYVVDLANVQRMFKAWRAAMPRVVPFYAVKCNPEPGILKLLNALGAGFDCASKGELDMMLRMGVSPNRIIFAHPCKRASDIRYAREHNVQYTTFDTVSELHKIAQMNPDFKCVLRIRADDPDARVPLGLKYGAEVSEADVLLRTAKELGLQVVGVSFHVGSACQNLSTFSGAIENARKVFDEAGALGFNMELLDIGGGFTGHFDEMGNVMFGEIANTINAALATNFPPEMGVRVIAEPGRYFAETSSTLLTPVYGQRDRVAADGSVKKDYWLTDGLYGSFNCILYDGQNPGYKVVR SPLMADSTDSRTFLSTLWGPTCDSADCVYKDVTLPVLRNGDWLMWNNAGAYTVAGACDFNGIEFTTPGKLYVWSDSAVDAAEEGADEQVMNA

**Isofrom 2: (335 nts of intron 5 starting at 222 position retained)**

ATGACAACTTCCGAGGTTCTGAGTCCCCCGCCCCCGAGCCAGGTGGCTAGCCTGGCTCAGCCG

CAGTTCAACGTGGAGGCGCTTCAGGCCTTCTCGATGCCCCTGGTGGCCGACGTTGTCAACACTGAAAATGGCGACGTCGCTTCGTTCGCCCGCAGCTCCTGGCACAAGGAGTCGGGCAAGGCGGGGGCGTTCGGCCTGCCGACCATGCTGGATATGAAGGCTGAGGCTCTGGAGCAGGTGCAGCTGGACACCTGGTCGGCGCAGCCTGTGGACCGTGGTCCGGCAGCAGCGGCTCCTGACCCCAAGCTGCTGCGCTCCTGGCAGTACCAGGCTACTGGCAAGCTTGCAACCGCTCAGGTCCTGGTGGACGTCAAGGCTGAGCACATCAAGGCTGGTGGCCCCATGGGCATGAAGCAGGAGGCCATTAAGCACATCCTGCGCCACAAGCCGGACGACACCTTCTATGTGGTTGACCTGGCTAACGTGCAGCGCATGTTCAAGGCTAGCCCTGCCTGACTTTTCATTCGCAACCATTAGGCATGTGCCCCCACACATCCCCACCACACCACCACCACCACGCGCCCGGCATCGCGTCATCTACATCCACGCCTGCATCAAGCTGCTCTGCTCGGCCTGGAGTCATCCGGGCCTTGCCATCAGCGCCAAGTGCACGTCAACGCACACGCCTCACCAAGCGCAAGCCTTCTCCTTCATGGGCAAGGTCTTAAGCGGTGGTGGTGGTGGTGGCGGCTGGCACCTGGAGACGGAGCGTGGAGCGTGGAGCGGAGCCCGAAGACCCAGCATCGTTGCAAACAGCACAACCCCTGTTGCTCCTGGCCTGGCGCGCCGCTATGCCCCGCGTGGTCCCCTTCTACGCGGTCAAGTGCAACCCGGAGCCGGGTATCCTGAAGCTGCTGAACGCGCTGGGTGCTGGCTTCGACTGCGCTAGCAAGGGCGAGCTGGACATGATGCTGCGCATGGGCGTCTCGCCCAACCGCATTATCTTTGCTCACCCCTGCAAGCGCGCCTCGGACATCCGCTACGCGCGCGAGCACAACGTG

CAGTACACCACGTTCGACACCGTGTCGGAGCTGCACAAGATCGCGCAGATGAACCCCGACTTCAAGTGCGTGCTGCGCATCCGCGCCGACGACCCGGACGCCCGCGTGCCTCTGGGCCTGAAGTACGGCGCTGAGGTGTCGGAGGCCGACGTGCTGCTGCGCACGGCGAAGGAGCTGGGCCTGCAGGTGGTGGGCGTCAGCTTCCACGTTGGCAGCGCTTGCCAGAACCTGTCCACCTTCTCGGGCGCCATTGAGAACGCGCGCAAGGTGTTCGACGAGGCCGGCGCCCTGGGCTTCAACATGGAGCTGCTGGATATCGGCGGTGGCTTCACCGGCCACTTCGACGAGATGGGCAACGTGATGTTCGGCGAGATCGCGAACACCATCAACGCCGCTCTGGCCACCAACTTCCCGCCGGAGATGGGCGTGCGCGTCATCGCCGAGCCCGGCCGCTACTTCGCTGAGACCAGCAGCACGCTGCTGACCCCCGTCTACGGCCAGCGCGACCGCGTGGCTGCCGACGGCTCCGTGAAGAAGGACTACTGGCTCACCGACGGCCTGTACGGCTCCTTCAACTGCATTCTGTACGACGGCCAGAACCCTGGCTACAAGGTCGTGCGCTCGCCGCTGATGGCCGACTCGACCGACAGCCGCACCTTCCTGTCGACCCTGTGGGGTCCCACCTGCGACAGCGCTGACTGCGTCTACAAGGACGTGACCCTGCCCGTGCTGCGCAACGGTGACTGGCTGATGTGGAACAACGCCGGCGCCTACACCGTGGCCGGTGCCTGCGACTTCAACGGCATCGAGTTCACCACTCCGGGCAAGCTGTACGTGTGGTCCGACAGCGCGGTGGACGCGGCCGAGGAGGGCGCTGACGAGCAGGTCATGAACGCGTGA

**Isoform 2 protein: (172 aa)**

MTTSEVLSPPPPSQVASLAQPQFNVEALQAFSMPLVADVVNTENGDVASFARSSWHKESGKAGAFGLPTMLDMKAEALEQVQLDTWSAQPVDRGPAAAAPDPKLLRSWQYQATGKLATAQVLVDVKAEHIKAGGPMGMKQEAIKHILRHKPDDTFYVVDLANVQRMFKASPA

**Isofrom 3: (intron 5 starting at 222 position retained);**

ATGACAACTTCCGAGGTTCTGAGTCCCCCGCCCCCGAGCCAGGTGGCTAGCCTGGCTCAGCCG

CAGTTCAACGTGGAGGCGCTTCAGGCCTTCTCGATGCCCCTGGTGGCCGACGTTGTCAACACTGAAAATGGCGACGTCGCTTCGTTCGCCCGCAGCTCCTGGCACAAGGAGTCGGGCAAGGCGGGGGCGTTCGGCCTGCCGACCATGCTGGATATGAAGGCTGAGGCTCTGGAGCAGGTGCAGCTGGACACCTGGTCGGCGCAGCCTGTGGACCGTGGTCCGGCAGCAGCGGCTCCTGACCCCAAGCTGCTGCGCTCCTGGCAGTACCAGGCTACTGGCAAGCTTGCAACCGCTCAGGTCCTGGTGGACGTCAAGGCTGAGCACATCAAGGCTGGTGGCCCCATGGGCATGAAGCAGGAGGCCATTAAGCACATCCTGCGCCACAAGCCGGACGACACCTTCTATGTGGTTGACCTGGCTAACGTGCAGCGCATGTTCAAGGCTAGCCCTGCCTGACTTTTCATTCGCAACCATTAGGCATGTGCCCCCACACATCCCCACCACACCACCACCACCACGCGCCCGGCATCGCGTCATCTACATCCACGCCTGCATCAAGCTGCTCTGCTCGGCCTGGAGTCATCCGGGCCTTGCCATCAGCGCCAAGTGCACGTCAACGCACACGCCTCACCAAGCGCAAGCCTTCTCCTTCATGGGCAAGGTCTTAAGCGGTGGTGGTGGTGGTGGCGGCTGGCACCTGGAGACGGAGCGTGGAGCGTGGAGCGGAGCCCGAAGACCCAGCATCGTTGCAAACAGCACAACCCCTGTTGCTCCTGGTAACCCTCTCGCATACTCACGCGCCATACTAATTGGGCCCGTTGTCTCTCTCTTGTCTTAGCAGCTAGGTGTCTACTGGGCGCCATATGCACCGCATGAGTCTTTGTGCCTTGTGCCGTTCGCGTGTGGCGTGTTGAGCATGCGCCTGCGTGTACGCGTGTTTGTC

TGTGCGGTTTTTTTGTGCCAGTCCGAGTGAGTCCTGGGCACGTGACCGCTCACATGGCTCCTGGACCCAGCGCGCGTTAATCGCTGGCGCGCCCCCCTATGGGGCGCGTTGATGCTTCGCGTTGCATGGACTAGGTAGCATTGTGCCTCTGTCTTGTCGTTGTCTCGCGTGTGCACACGCATGAAGCCACGTGGCGACCTCCTGGTCTACAAAATCCATCAGACCAGAGAGCGCGCGCCATCACGGCCAG

GAGGGCCCGTGGCTACCTTCGGCGCCTTCTGATTTCCCAACTTGTCCCATCAAGTTGGGATGGAAGGAATTGCCAACTCCAGGTGGTTGCTGACGTTGTGCGTTCCGTTTTCTCCTCTCCCTCAGGCCTGGCGCGCCGCTATGCCCCGCGTGGTCCCCTTCTACGCGGTCAAGTGCAACCCGGAGCCGGGTATCCTGAAGCTGCTGAACGCGCTGGGTGCTGGCTTCGACTGCGCTAGCAAGGGCGAGCTGGACATGATGCTGCGCATGGGCGTCTCGCCCAACCGCATTATCTTTGCTCACCCCTGCAAGCGCGCCTCGGACATCCGCTACGCGCGCGAGCACAACGTGCAGTACACCACGTTCGACACCGTGTCGGAGCTGCACAAGATCGCGCAGATGAACCCCGACTTCAAGTGCGTGCTGCGCATCCGCGCCGACGACCCGGACGCCCGCGTGCCTCTGGGCCTGAAGTACGGCGCTGAGGTGTCGGAGGCCGACGTGCTGCTGCGCACGGCGAAGGAGCTGGGCCTGCAGGTGGTGGGCGTCAGCTTCCACGTTGGCAGCGCTTGCCAGAACCTGTCCACCTTCTCGGGCGCCATTGAGAACGCGCGCAAGGTGTTCGACGAGGCCGGCGCCCTGGGCTTCAACATGGAGCTGCTGGATATCGGCGGTGGCTTCACCGGCCACTTCGACGAGATGGGCAACGTGATGTTCGGCGAGATCGCGAACACCATCAACGCCGCTCTGGCCACCAACTTCCCGCCGGAGATGGGCGTGCGCGTCATCGCCGAGCCCGGCCGCTACTTCGCTGAGACCAGCAGCACGCTGCTGACCCCCGTCTACGGCCAGCGCGACCGCGTGGCTGCCGACGGCTCCGTGAAGAAGGACTACTGGCTCACCGACGGCCTGTACGGCTCCTTCAACTGCATTCTGTACGACGGCCAGAACCCTGGCTACAAGGTCGTGCGCTCGCCGCTGATGGCCGACTCGACCGACAGCCGCACCTTCCTGTCGACCCTGTGGGGTCCCACCTGCGACAGCGCTGACTGCGTCTACAAGGACGTGACCCTGCCCGTGCTGCGCAACGGTGACTGGCTGATGTGGAACAACGCCGGCGCCTACACCGTGGCCGGTGCCTGCGACTTCAACGGCATCGAGTTCACCACTCCGGGCAAGCTGTACGTGTGGTCCGACAGCGCGGTGGACGCGGCCGAGGAGGGCGCTGACGAGCAGGTCATGAACGCGTGA

**Isoform 3 protein: (172aa)**

MTTSEVLSPPPPSQVASLAQPQFNVEALQAFSMPLVADVVNTENGDVASFARSSWHKESGKAGAFGLPTMLDMKAEALEQVQLDTWSAQPVDRGPAAAAPDPKLLRSWQYQATGKLATAQVLVDVKAEHIKAGGPMGMKQEAIKHILRHKPDDTFYVVDLANVQRMFKASPA

**Isoform 4: (first 557 bases of 5^th^ intron retained)**

ATGACAACTTCCGAGGTTCTGAGTCCCCCGCCCCCGAGCCAGGTGGCTAGCCTGGCTCAGCCG

CAGTTCAACGTGGAGGCGCTTCAGGCCTTCTCGATGCCCCTGGTGGCCGACGTTGTCAACACTGAAAATGGCGACGTCGCTTCGTTCGCCCGCAGCTCCTGGCACAAGGAGTCGGGCAAGGCGGGGGCGTTCGGCCTGCCGACCATGCTGGATATGAAGGCTGAGGCTCTGGAGCAGGTGCAGCTGGACACCTGGTCGGCGCAGCCTGTGGACCGTGGTCCGGCAGCAGCGGCTCCTGACCCCAAGCTGCTGCGCTCCTGGCAGTACCAGGCTACTGGCAAGCTTGCAACCGCTCAGGTCCTGGTGGACGTCAAGGCTGAGCACATCAAGGCTGGTGGCCCCATGGGCATGAAGCAGGAGGCCATTAAGCACATCCTGCGCCACAAGCCGGACGACACCTTCTATGTGGTTGACCTGGCTAACGTGCAGCGCATGTTCAAGGTAATGAATTGATCCTCGCCGTCGCGACTACCAGCGCGCGCGAGCGGGGTGGGTCGGGACCATGGTTGGGTTTCGTTCATGCCTGTGGGTTGGGCTGCGCCGTGCATGCCATGGCGCCTGGTTGCGTAGCGGAGCGCGCGAGAGCGAGCGGCACGGCGAATGAGCATGCGCAATTCCGAATTAACTCGGCCCTGGTGTGCTGTCCTATGTTCGACCTCGCAGGCTAGCCCTGCCTGACTTTTCATTCGCAACCATTAGGCATGTGCCCCCACACATCCCCACCACACCACCACCACCACGCGCCCGGCATCGCGTCATCTACATCCACGCCTGCATCAAGCTGCTCTGCTCGGCCTGGAGTCATCCGGGCCTTG

CCATCAGCGCCAAGTGCACGTCAACGCACACGCCTCACCAAGCGCAAGCCTTCTCCTTCATGGGCAAGGTCTTAAGCGGTGGTGGTGGTGGTGGCGGCTGGCACCTGGAGACGGAGCGTGGAGCGTGGAGCGGAGCCCGAAGACCCAGCATCGTTGCAAACAGCACAACCCCTGTTGCTCCTGGCCTGGCGCGCCGCTATGCCCCGCGTGGTCCCCTTCTACGCGGTCAAGTGCAACCCGGAGCCGGGTATCCTGAAGCTGCTGAACGCGCTGGGTGCTGGCTTCGACTGCGCTAGCAAGGGCGAGCTGGACATGATGCTGCGCATGGGCGTCTCGCCCAACCGCATTATCTTTGCTCACCCCTGCAAGCGCGCCTCGGACATCCGCTACGCGCGCGAGCACAACGTGCAGTACACCACGTTCGACACCGTGTCGGAGCTGCACAAGATCGCGCAGATGAACCCCGACTTCAAGTGCGTGCTGCGCATCCGCGCCGACGACCCGGACGCCCGCGTGCCTCTGGGCCTGAAGTACGGCGCTGAGGTGTCGGAGGCCGACGTGCTGCTGCGCACGGCGAAGGAGCTGGGCCTGCAGGTGGTGGGCGTCAGCTTCCACGTTGGCAGCG

CTTGCCAGAACCTGTCCACCTTCTCGGGCGCCATTGAGAACGCGCGCAAGGTGTTCGACGAGGCCGGCGCCCTGGGCTTCAACATGGAGCTGCTGGATATCGGCGGTGGCTTCACCGGCCACTTCGACGAGATGGGCAACGTGATGTTCGGCGAGATCGCGAACACCATCAACGCCGCTCTGGCCACCAACTTCCCGCCGGAGATGGGCGTGCGCGTCATCGCCGAGCCCGGCCGCTACTTCGCTGAGAC

CAGCAGCACGCTGCTGACCCCCGTCTACGGCCAGCGCGACCGCGTGGCTGCCGACGGCTCCGTGAAGAAGGACTACTGGCTCACCGACGGCCTGTACGGCTCCTTCAACTGCATTCTGTACGACGGCCAGAACCCTGGCTACAAGGTCGTGCGCTCGCCGCTGATGGCCGACTCGACCGACAGCCGCACCTTCCTGTCGACCCTGTGGGGTCCCACCTGCGACAGCGCTGACTGCGTCTACAAGGACGTG

ACCCTGCCCGTGCTGCGCAACGGTGACTGGCTGATGTGGAACAACGCCGGCGCCTACACCGTGGCCGGTGCCTGCGACTTCAACGGCATCGAGTTCACCACTCCGGGCAAGCTGTACGTGTGGTCCGACAGCGCGGTGGACGCGGCCGAGGAGGGCGCTGACGAGCAGGTCATGAACGCGTGA

**Isoform 4 protein: (171 aa)**

MTTSEVLSPPPPSQVASLAQPQFNVEALQAFSMPLVADVVNTENGDVASFARSSWHKESGKAGAFGLPTMLDMKAEALEQVQLDTWSAQPVDRGPAAAAPDPKLLRSWQYQATGKLATAQVLVDVKAEHIKAGGPMGMKQEAIKHILRHK PDDTFYVVDLANVQRMFKVMN

**Isoform 5: (5^th^ intron retained)**

ATGACAACTTCCGAGGTTCTGAGTCCCCCGCCCCCGAGCCAGGTGGCTAGCCTGGCTCAGCCG

CAGTTCAACGTGGAGGCGCTTCAGGCCTTCTCGATGCCCCTGGTGGCCGACGTTGTCAACACTGAAAATGGCGACGTCGCTTCGTTCGCCCGCAGCTCCTGGCACAAGGAGTCGGGCAAGGCGGGGGCGTTCGGCCTGCCGACCATGCTGGATATGAAGGCTGAGGCTCTGGAGCAGGTGCAGCTGGACACCTGGTCGGCGCAGCCTGTGGACCGTGGTCCGGCAGCAGCGGCTCCTGACCCCAAGCTGCTGCGCTCCTGGCAGTACCAGGCTACTGGCAAGCTTGCAACCGCTCAGGTCCTGGTGGACGTCAAGGCTGAGCACATCAAGGCTGGTGGCCCCATGGGCATGAAGCAGGAGGCCATTAAGCACATCCTGCGCCACAAGCCGGACGACACCTTCTATGTGGTTGACCTGGCTAACGTGCAGCGCATGTTCAAGGTAATGAATTGATCCTCGCCGTCGCGACTACCAGCGCGCGCGAGCGGGGTGGGTCGGGACCATGGTTGGGTTTCGTTCATGCCTGTGGGTTGGGCTGCGCCGTGCATGCCATGGCGCCTGGTTGCGTAGCGGAGCGCGCGAGAGCGAGCGGCACGGCGAATGAGCATGCGCAATTCCGAATTAACTCGGCCCTGGTGTGCTGTCCTATGTTCGACCTCGCAGGCTAGCCCTGCCTGACTTTTCATTCGCAACCATTAGGCATGTGCCCCCACACATCCCCACCACACCACCACCACCACGCGCCCGGCATCGCGTCATCTACATCCACGCCTGCATCAAGCTGCTCTGCTCGGCCTGGAGTCATCCGGGCCTTG

CCATCAGCGCCAAGTGCACGTCAACGCACACGCCTCACCAAGCGCAAGCCTTCTCCTTCATGGGCAAGGTCTTAAGCGGTGGTGGTGGTGGTGGCGGCTGGCACCTGGAGACGGAGCGTGGAGCGTGGAGCGGAGCCCGAAGACCCAGCATCGTTGCAAACAGCACAACCCCTGTTGCTCCTGGTAACCCTCTCGCATACTCACGCGCCATACTAATTGGGCCCGTTGTCTCTCTCTTGTCTTAGCAGCT

AGGTGTCTACTGGGCGCCATATGCACCGCATGAGTCTTTGTGCCTTGTGCCGTTCGCGTGTGGCGTGTTGAGCATGCGCCTGCGTGTACGCGTGTTTGTCTGTGCGGTTTTTTTGTGCCAGTCCGAGTGAGTCCTGGGCACGTGACCGCTCACATGGCTCCTGGACCCAGCGCGCGTTAATCGCTGGCGCGCCCCCCTATGGGGCGCGTTGATGCTTCGCGTTGCATGGACTAGGTAGCATTGTGCCTCTGTCTTGTCGTTGTCTCGCGTGTGCACACGCATGAAGCCACGTGGCGACCTCCTGGTCTACAAAATCCATCAGACCAGAGAGCGCGCGCCATCACGGCCAGGAGGGCCCGTGGCTACCTTCGGCGCCTTCTGATTTCCCAACTTGTCCCATCAAGTTGGGATGGAAGGAATTGCCAACTCCAGGTGGTTGCTGACGTTGTGCGTTCCGTTTTCTCCTCTCCCTCAGGCCTGGCGCGCCGCTATGCCCCGCGTGGTCCCCTTCTACGCGGTCAAGTGCAACCCGGAGCCGGGTATCCTGAAGCTGCTGAACGCGCTGGGTGCTGGCTTCGACTGCGCTAGCAAGGGCGAGCTGGACATGATGCTGCGCATGGGCGTCTCGCCCAACCGCATTATCTTTGCTCACCCCTGCAAGCGCGCCTCGGACATCCGCTACGCGCGCGAGCACAACGTGCAGTACACCACGTTCGACACCGTGTCGGAGCTGCACAAGATCGCGCAGATGAACCCCGACTTCAAGTGCGTGCTGCGCATCCGCGCCGACGACCCGGACGCCCGCGTGCCTCTGGGC

CTGAAGTACGGCGCTGAGGTGTCGGAGGCCGACGTGCTGCTGCGCACGGCGAAGGAGCTGGGCCTGCAGGTGGTGGGCGTCAGCTTCCACGTTGGCAGCGCTTGCCAGAACCTGTCCACCTTCTCGGGCGCCATTGAGAACGCGCGCAAGGTGTTCGACGAGGCCGGCGCCCTGGGCTTCAACATGGAGCTGCTGGATATCGGCGGTGGCTTCACCGGCCACTTCGACGAGATGGGCAACGTGATGTTCG

GCGAGATCGCGAACACCATCAACGCCGCTCTGGCCACCAACTTCCCGCCGGAGATGGGCGTGCGCGTCATCGCCGAGCCCGGCCGCTACTTCGCTGAGACCAGCAGCACGCTGCTGACCCCCGTCTACGGCCAGCGCGACCGCGTGGCTGCCGACGGCTCCGTGAAGAAGGACTACTGGCTCACCGACGGCCTGTACGGCTCCTTCAACTGCATTCTGTACGACGGCCAGAACCCTGGCTACAAGGTCGT

GCGCTCGCCGCTGATGGCCGACTCGACCGACAGCCGCACCTTCCTGTCGACCCTGTGGGGTCCCACCTGCGACAGCGCTGACTGCGTCTACAAGGACGTGACCCTGCCCGTGCTGCGCAACGGTGACTGGCTGATGTGGAACAACGCCGGCGCCTACACCGTGGCCGGTGCCTGCGACTTCAACGGCATCGAGTTCACCACTCCGGGCAAGCTGTACGTGTGGTCCGACAGCGCGGTGGACGCGGCCGAGGAGGGCGCTGACGAGCAGGTCATGAACGCGTGA

**Isofrom 5 protein: (171 aa)**

MTTSEVLSPPPPSQVASLAQPQFNVEALQAFSMPLVADVVNTENGDVASFARSSWHKESGKAGAFGLPTMLDMKAEALEQVQLDTWSAQPVDRGPAAAAPDPKLLRSWQYQATGKLATAQVLVDVKAEHIKAGGPMGMKQEAIKHILRHKPDDTFYVVDLANVQRMFKVMN

**Isofrom 6: (all introns retained)**

ATGACAACTTCCG

AGGTTCTGAGTCCCCCGCCCCCGAGCCAGGTGGCTAGCCTGGCTCAGCCG

CAGTTCAACGTGGAGGCGCTTCAGGCCTTCTCGATGCCCCTGGTGGCCGA

CGTTGTCAACACTGAAAATGGCGACGTCGCTTCGTTCGCCCGCAGCTCCT

GGCACAAGGAGTCGGGCAAGGCGGGGGCGGTGAGTTTTCAGAGTTTGACG

ATCGCGGAATTCCCCGGTTGTCGGCTGCCTTCGGCCTTGGGCCAGCGCCC

CGCGCGCTTGTACTTGTGTGGCACACATTTAGTGGTTTGGGGGACCGGAT

CCGGTGATCGGCCGAGCGTCTGTCACCAGCCCATCAGGCCCTCGTGGCCT

TTGCTAAGCGCCGCTCGCGATCTGGCCTCATTTCCTAACCCAATTCTCTC

TGCATGTGCGCGCAGTTCGGCCTGCCGACCATGCTGGATATGAAGGCTGA

GGCTCTGGAGCAGGTGCAGCTGGACACCTGGTCGGCGCAGCCTGTGGACC

GTGGTCCGGCAGCAGCGGCTCCTGACCCCAAGCTGCTGCGCTCCTGGCAG

TACCAGGCTACTGGCAAGCTTGCAACCGCTCAGGTCCTGGTGGACGTCAA

GGCTGAGCACATCAAGGCTGGTGAGTTCCTAGGCTCACGTGGGACTGGGG

GCAGACCGTAGACGCAGACTGGCTAAGGAGTCTGGCTGCCGTTCCAGGCG

AAACGAGGTGCATGTGGGTTTGCCGGACCCCGTTGTGAGTGTGTGATCAG

TGAAGTCAGCCATCTACAAATTTGTAAGATTGTCGACTGATTATTGCTGT

TTTTTCTCTTCCCACGTAGGTGGCCCCATGGGCATGAAGCAGGAGGCCAT

TAAGCACATCCTGCGCCACAAGCCGGACGACACCTTCTATGTGGTTGACC

TGGCTAACGTGCAGCGCATGTTCAAGGTAATGAATTGATCCTCGCCGTCG

CGACTACCAGCGCGCGCGAGCGGGGTGGGTCGGGACCATGGTTGGGTTTC

GTTCATGCCTGTGGGTTGGGCTGCGCCGTGCATGCCATGGCGCCTGGTTG

CGTAGCGGAGCGCGCGAGAGCGAGCGGCACGGCGAATGAGCATGCGCAAT

TCCGAATTAACTCGGCCCTGGTGTGCTGTCCTATGTTCGACCTCGCAGGC

TAGCCCTGCCTGACTTTTCATTCGCAACCATTAGGCATGTGCCCCCACAC

ATCCCCACCACACCACCACCACCACGCGCCCGGCATCGCGTCATCTACAT

CCACGCCTGCATCAAGCTGCTCTGCTCGGCCTGGAGTCATCCGGGCCTTG

CCATCAGCGCCAAGTGCACGTCAACGCACACGCCTCACCAAGCGCAAGCC

TTCTCCTTCATGGGCAAGGTCTTAAGCGGTGGTGGTGGTGGTGGCGGCTG

GCACCTGGAGACGGAGCGTGGAGCGTGGAGCGGAGCCCGAAGACCCAGCA

TCGTTGCAAACAGCACAACCCCTGTTGCTCCTGGTAACCCTCTCGCATAC

TCACGCGCCATACTAATTGGGCCCGTTGTCTCTCTCTTGTCTTAGCAGCT

AGGTGTCTACTGGGCGCCATATGCACCGCATGAGTCTTTGTGCCTTGTGC

CGTTCGCGTGTGGCGTGTTGAGCATGCGCCTGCGTGTACGCGTGTTTGTC

TGTGCGGTTTTTTTGTGCCAGTCCGAGTGAGTCCTGGGCACGTGACCGCT

CACATGGCTCCTGGACCCAGCGCGCGTTAATCGCTGGCGCGCCCCCCTAT

GGGGCGCGTTGATGCTTCGCGTTGCATGGACTAGGTAGCATTGTGCCTCT

GTCTTGTCGTTGTCTCGCGTGTGCACACGCATGAAGCCACGTGGCGACCT

CCTGGTCTACAAAATCCATCAGACCAGAGAGCGCGCGCCATCACGGCCAG

GAGGGCCCGTGGCTACCTTCGGCGCCTTCTGATTTCCCAACTTGTCCCAT

CAAGTTGGGATGGAAGGAATTGCCAACTCCAGGTGGTTGCTGACGTTGTG

CGTTCCGTTTTCTCCTCTCCCTCAGGCCTGGCGCGCCGCTATGCCCCGCG

TGGTCCCCTTCTACGCGGTCAAGTGCAACCCGGAGCCGGGTATCCTGAAG

CTGCTGAACGCGCTGGGTGCTGGCTTCGACTGCGCTAGCAAGGGCGAGCT

GGACATGATGCTGCGCATGGGCGTCTCGCCCAACCGCATTATCTTTGCTC

ACCCCTGCAAGCGCGCCTCGGACATCCGCTACGCGCGCGAGCACAACGTG

CAGTACACCACGGTGAGTGTGCTGGGGCTGGGGAATGGGCAGTGGGACGG

CACTCGCTTCTGGGTCCAAGCATTGTATTTGTGGCAAGGAAAGCTCTTCA

CTGCAACGCTGACTTGTTACGTTGCCTTGCACCGTTTACCCGCAGTTCGA

CACCGTGTCGGAGCTGCACAAGATCGCGCAGATGAACCCCGACTTCAAGT

GCGTGCTGCGCATCCGCGCCGACGACCCGGACGCCCGCGTGCCTCTGGGC

CTGAAGTACGGCGCTGAGGTGTCGGAGGCCGACGTGCTGCTGCGCACGGC

GAAGGAGCTGGGCCTGCAGGTGGTGGGCGTCAGCTTCCACGTTGGCAGCG

CTTGCCAGAACCTGTCCACCTTCTCGGGCGCCATTGAGAACGCGCGCAAG

GTGTTCGACGAGGCCGGCGCCCTGGGCTTCAACATGGAGCTGCTGGATAT

CGGCGGTGGCTTCACCGGCCACTTCGACGAGATGGGCAACGTGATGTTCG

GCGAGATCGCGAACACCATCAACGCCGCTCTGGCCACCAACTTCCCGCCG

GAGATGGGCGTGCGCGTCATCGCCGAGCCCGGCCGCTACTTCGCTGAGAC

CAGCAGCACGCTGCTGACCCCCGTCTACGGCCAGCGCGACCGCGTGGCTG

CCGACGGCTCCGTGAAGAAGGACTACTGGCTCACCGACGGCCTGTACGGC

TCCTTCAACTGCATTCTGTACGACGGCCAGAACCCTGGCTACAAGGTCGT

GCGCTCGCCGCTGATGGCCGACTCGACCGACAGCCGCACCTTCCTGTCGA

CCCTGTGGGGTCCCACCTGCGACAGCGCTGACTGCGTCTACAAGGACGTG

ACCCTGCCCGTGCTGCGCAACGGTGACTGGCTGATGTGGAACAACGCCGG

CGCCTACACCGTGGCCGGTGCCTGCGACTTCAACGGCATCGAGTTCACCA

CTGTGAGTTTGCCATCAACCATCGGCTCTTTTGTTCGGTCGGGTGCACGG

TGTAAGGGTCATTTGATGATTTATGCTGCTATCATCTGTACCGATATCCA

ACTGACCTCCAACTTGTCCTTTTGCGCGCAGCCGGGCAAGCTGTACGTGT

GGTCCGACAGCGCGGTGGACGCGGCCGAGGAGGGCGCTGACGAGCAGGTC

ATGAACGCGTGA

**Isoform 6 protein: (151 aa)**

MTTSEVLSPPPPSQVASLAQPQFNVEALQAFSMPLVADVVNTENGDVASFARSSWHKESGKAGAVSFQSLTIAEFPGCRLPSALGQRPARLYLCGTHLVVWGTGSGDRPSVCHQPIRPSWPLLSAARDLASFPNPILSACARSSACRPCWI

**B) ASyn splice variants**

**Genomic Clone (2989 bases)**

GCTCAGCAAGATAGTCAATCGACACAATACCTCAAAACCCCTTTCACGCAAAGCAGATTCGCGACATTTGCAAGCTCTCGAGGCTTCGGAACGGAGGGCGCCACCATGGTTCTGCACATGCTAGCAGTGTTTGGCCGCGAGACGGCCCACCCTCCTGTGTTGATCGAGTCGGAGGAGGCGCTCAAGAAGACACATGCGCCAATGTTAACGGACGAGGAGCAAGCGGCAGAGGACGTAAGTGGAAGGGCGTCTCGCGGCCGGTACCCTCCGACACGCCGCGATCGACCGGGACATTGTTTTTGTAATGTGTTTGGGCAATTGAACCGAATGCCATCGAGAAAGACCGCGTCGAGAGTTTGCTTGGTTCCGAGAATTCAGTCGCGATCCCGCCACCCTCTTCCACTGCAGGCTGCTTGCGCCAAGGTTATAGACACTGTATTCAACGCGGCAAAGCCCGCGTCAAGGCACAAGCAGCTGCTGGGCAAGGGCAGGTCAGAAAACAGCAGGCGGGGGACTGGGCCTGGCTGCGTGTGTATCGCTGACACGCCATACTCTGCTTTGGGGTCCGTCCGCCCCGCCAATCAATTTTATGGACGGCTAGATTCTGGAATAACCCACGCGCGCGTCCTGCCCTGCTCTCGTCCACCCGAACTCTTGTTCCCCCAACCCAACTGTTGCGTGTCCTCGCATCCCACGTGCATGTGATCCGCCACAGTGGATACCTGTACGAGGCGTCGCCCTACTGCTCGTACGCTGAGCGCGACGGCGTGCACGTCATCTTCACTGGGGAGGTCGGCGAGTGGCCCGGCATTGACGTCGTCTCTTCCGCCCACGACGGTAAGAGCAATCGCGGCTTCGGCGCATGCAACTCCTGTGAACAAACATTTGCGCCTACACTGCATACCTGCAGCCTTCGTCCGCAATGAGCCCCCGCTCGAGGCGAACGACGCCGCCTGGCTGCTCGACTTCTACGGGTAAGCAACGCACTTGCTTGCAGCTGACAAAGCAATCTGGCCCCGCATGAGCGTGACCCTCATTGGTCTTGGCCCGCTCAGCTCGGCAGCTTCCATGACGCTCCGCGCCCTTTGATCACCCCCTATGGCACAGGACGTTCGGCCGCGGCGCCAGCGAAAGCACCACGCAGCGCGCGCTGGAATGCCTGGCCCGTGTGAAGGGCACCTTCGCTTTCATCATCTACGATGCCGTGCACGTGAGTGAAGGCCGCGGCGAGCCCGCCGTTACACCTGGGGGTTGTCAGGGCCTAGCCGCATGCAATTCCCTCGAAGCCCCAGCGTACGCACCTGCGCAGCGCTCATCCTGGCCCCACGCCTTCCCTGTGCCCCAGTCTCGTCCTTTCCATCTGTACGCTCACGTCCTTTCGTCCACATGCCCTTCCTGCCACGCAGCACCGCGTGCTGGCCGCCCGCGACAGCGAGGGCGTGCAGCCGCTGTTCTGGGGCTGCACCGACAGCGGCCAGCTCATGTTCGGCAGCGTGGCCGACGACCTGGACGGATGCAACCCCACCGCCGCGCCCTTCCCCTCCGGCACCCTGTTCGCCAGGTGCGTTGGATGGGGAGCGCGCGCGCTTTCATGAGCAGGCCAGGGGGGTCGTGTTTGCTATAGAGTGGCAGCTGGAAGACCCAGCCATCCCTTGCTTGGACCTCAAGCCTTCGGCAGTGAACAGGCACAAGTGACGTGTACATTGCGAAAGGACAACGGAACGAGAACGGAGAACTGACGTGTGTTTGACGTGTCGCCTGACTGTCCATCCACTGCCACTTCCACAGTGAGCGGCACACGGTGGCGTACAGCCCCGGTGCTTACGGCTGGGTGATTGTGGATGATGACTTTCCCGGCCTCATCATGAGCTTCCAGCGCACCAAGGAGGGGGCAGAGGGCTGGCGCAACGTCAAGGTGGGTGGCGGGTGGTATGGGACTGGATGCATTGGCGGGTGAGGATGGGCGGCGGGTGCTTCTAAGATCGTAGCGCTGCAGAGGCGTGGGGTTGTTGTGTTGCAGTTTGGCATTGGGTCATGGGGAACCGCTCGTCGCTCAGTCTTGTAGCCAAGCCGGGACACGTTCCGACCACGGGGCTTCGTCATGTTGTCCGTGACTTTGTTATGCATGCCATCTGCGTTCATGCCGTTTGCCGCGCTCTCCTGCTGCAGGCCATTCCGCGCGTCACCAGCAAGGGCGTGGTGTGCGGCGCGGTCTACAAGGTGGCGAGCGCGCAGAACATCGACTCCGCCTAAAGGACGACCGCTTAGCAGTTGGAGGAGCAAAAACGGGGGCTCTGGACTGATGGAGGTCAGGTGGTTGGGCGTGTAGCAGAGGAGTTGCGCTGTGTGGCTGCTGTGTTCCACAAGGGCAGCGACGGGTGCAAAAACATGCATTACGAGGCTGGCTTGCTACGGTTTGCGTTCCGATGCAAGATGTTGATATTCTGGTTACGAAAGAGCCAGGTGCAAACGCAACCCTCATAAGGCATGTTGAATTGGCGGCAGTTGCGGCGCGATGTTTGGTTGACCGGATGCTGGTTGAAGGCTGGGCGGACGTGCCTGGGCTCGGATAACGGGTTGTTCCATTGTTTTGGACCTGCTGGACGGCTGTTTACACCCTGCTATCGATTGCTACTGCGGCGTAAGTGCCGTTAACGGCGCAGAGACAGGGGGCAGCTTTACACCCCCTGCGACTGGGGACTTCTGGAACATCTAGGTGATGCATGCGTAGCTCAGCCGCAAGGGGAGGTGCGTTCAAGTCCCTGACTCTGCAATGTGGAGTCCTGTTTACCGTGTGGCGTTATGCAAGCTTAAATGCGCAGCAGACACGAAGAGCTTCATTCAGGACTCGACAAGTGTTCGCCGGTCACAAGAAAAGGGGCTGAGCCCCTCCACAGTCTGAGGCCGAACAACCTCATCGCCCGGACATACGGTAGCCGGGGTCGGTTTCCACAGGCACATAGCCAAACCATGTGGCTATCTAGCAGTC

**Isoform 1 (867 bases)**

ATGGTTCTGCACATGCTAGCAGTGTTTGGCCGCGAGACGGCCCACCCTCCTGTGTTGATCGAGTCGGAGGAGGCGCTCAAGAAGACACATGCGCCAATGTTAACGGACGAGGAGCAAGCGGCAGAGGACGCTGCTTGCGCCAAGGTTATAGACACTGTATTCAACGCGGCAAAGCCCGCGTCAAGGCACAAGCAGCTGCTGGGCAAGGGCAGTGGATACCTGTACGAGGCGTCGCCCTACTGCTCGTACGCTGAGCGCGACGGCGTGCACGTCATCTTCACTGGGGAGGTCGGCGAGTGGCCCGGCATTGACGTCGTCTCTTCCGCCCACGACGCCTTCGTCCGCAATGAGCCCCCGCTCGAGGCGAACGACGCCGCCTGGCTGCTCGACTTCTACGGGACGTTCGGCCGCGGCGCCAGCGAAAGCACCACGCAGCGCGCGCTGGAATGCCTGGCCCGTGTGAAGGGCACCTTCGCTTTCATCATCTACGATGCCGTGCACCACCGCGTGCTGGCCGCCCGCGACAGCGAGGGCGTGCAGCCGCTGTTCTGGGGCTGCACCGACAGCGGCCAGCTCATGTTCGGCAGCGTGGCCGACGACCTGGACGGATGCAACCCCACCGCCGCGCCCTTCCCCTCCGGCACCCTGTTCGCCAGTGAGCGGCACACGGTGGCGTACAGCCCCGGTGCTTACGGCTGGGTGATTGTGGATGATGACTTTCCCGGCCTCATCATGAGCTTCCAGCGCACCAAGGAGGGGGCAGAGGGCTGGCGCAACGTCAAGGCCATTCCGCGCGTCACCAGCAAGGGCGTGGTGTGCGGCGCGGTCTACAAGGTGGCGAGCGCGCAGAACATCGACTCCGCCTAA

**Isoform 1 Protein: (288 aa)**

MVLHMLAVFGRETAHPPVLIESEEALKKTHAPMLTDEEQAAEDAACAKVIDTVFNAAKPASRHKQLLGKGSGYLYEASPYCSYAERDGVHVIFTGEVGEWPGIDVVSSAHDAFVRNEPPLEANDAAWLLDFYGTFGRGASESTTQRALECLARVKGTFAFIIYDAVHHRVLAARDSEGVQPLFWGCTDSGQLMFGSVADDLDGCNPTAAPFPSGTLFASERHTVAYSPGAYGWVIVDDDFPGLIMSFQRTKEGAEGWRNVKAIPRVTSKGVVCGAVYKVASAQNIDSA

**Isoform 2 (518 bases) (Exons 5 and 6 skipped and only 10bp of exon 4 retained)**

ATGGTTCTGCACATGCTAGCAGTGTTTGGCCGCGAGACGGCCCACCCTCCTGTGTTGATCGAGTCGGAGGAGGCGCTCAAGAAGACACATGCGCCAATGTTAACGGACGAGGAGCAAGCGGCAGAGGACGCTGCTTGCGCCAAGGTTATAGACACTGTATTCAACGCGGCAAAGCCCGCGTCAAGGCACAAGCAGCTGCTGGGCAAGGGCAGTGGATACCTGTACGAGGCGTCGCCCTACTGCTCGTACGCTGAGCGCGACGGCGTGCACGTCATCTTCACTGGGGAGGTCGGCGAGTGGCCCGGCATTGACGTCGTCTCTTCCGCCCACGACGCCTTCGTCCGCAGTGAGCGGCACACGGTGGCGTACAGCCCCGGTGCTTACGGCTGGGTGATTGTGGATGATGACTTTCCCGGCCTCATCATGAGCTTCCAGCGCACCAAGGAGGGGGCAGAGGGCTGGCGCAACGTCAAGGCCATTCCGCGCGTCACCAGCAAGGGCGTGGTGTGCGGCGCGGTCTACAAGGTGGCGAGCGCGCAGAACATCGACTCCGCCTAA

**Isoform 2 Protein: (185 aa)**

MVLHMLAVFGRETAHPPVLIESEEALKKTHAPMLTDEEQAAEDAACAKVIDTVFNAAKPASRHKQLLGKGSGYLYEASPYCSYAERDGVHVIFTGEVGEWPGIDVASSAHDAFVRSERHTVAYSPGAYGWVIVDDDFPGLIMSFQRTKEGAEGWRNVKAIPRVTSKGVVCGAVYKVASAQNIDSA

**Isoform 3 (745 bases) (Exon 4 skipped)**

ATGGTTCTGCACATGCTAGCAGTGTTTGGCCGCGAGACGGCCCACCCTCCTGTGTTGATCGAGTCGGAGGAGGCGCTCAAGAAGACACATGCGCCAATGTTAACGGACGAGGAGCAAGCGGCAGAGGACGCTGCTTGCGCCAAGGTTATAGACACTGTATTCAACGCGGCAAAGCCCGCGTCAAGGCACAAGCAGCTGCTGGGCAAGGGCAGCCTTCGTCCGCAATGAGCCCCCGCTCGAGGCGAACGACGCCGCCTGGCTGCTCGACTTCTACGGGACGTTCGGCCGCGGCGCCAGCGAAAGCACCACGCAGCGCGCGCTGGAATGCCTGGCCCGTGTGAAGGGCACCTTCGCTTTCATCATCTACGATGCCGTGCACCACCGCGTGCTGGCCGCCCGCGACAGCGAGGGCGTGCAGCCGCTGTTCTGGGGCTGCACCGACAGCGGCCAGCTCATGTTCGGCAGCGTGGCCGACGACCTGGACGGATGCAACCCCACCGCCGCGCCCTTCCCCTCCGGCACCCTGTTCGCCAGTGAGCGGCACACGGTGGCGTACAGCCCCGGTGCTTACGGCTGGGTGATTGTGGATGATGACTTTCCCGGCCTCATCATGAGCTTCCAGCGCACCAAGGAGGGGGCAGAGGGCTGGCGCAACGTCAAGGCCATTCCGCGCGTCACCAGCAAGGGCGTGGTGTGCGGCGCGGTCTACAAGGTGGCGAGCGCGCAGAACATCGACTCCGCCTAA

**Isoform 3 Protein: (75 aa)**

MVLHMLAVFGRETAHPPVLIESEEALKKTHAPMLTDEEQAAEDAACAKVIDTVFNAAKPASRHKQLLGKGSLRPQ

**Isoform 4 (928 bases) (61bp – Part of intron 5 is retained)**

ATGGTTCTGCACATGCTAGCAGTGTTTGGCCGCGAGACGGCCCACCCTCCTGTGTTGATCGAGTCGGAGGAGGCGCTCAAGAAGACACATGCGCCAATGTTAACGGACGAGGAGCAAGCGGCAGAGGACGCTGCTTGCGCCAAGGTTATAGACACTGTATTCAACGCGGCAAAGCCCGCGTCAAGGCACAAGCAGCTGCTGGGCAAGGGCAGTGGATACCTGTACGAGGCGTCGCCCTACTGCTCGTACGCTGAGCGCGACGGCGTGCACGTCATCTTCACTGGGGAGGTCGGCGAGTGGCCCGGCATTGACGTCGTCTCTTCCGCCCACGACGCCTTCGTCCGCAATGAGCCCCCGCTCGAGGCGAACGACGCCGCCTGGCTGCTCGACTTCTACGGGACGTTCGGCCGCGGCGCCAGCGAAAGCACCACGCAGCGCGCGCTGGAATGCCTGGCCCGTGTGAAGGGCACCTTCGCTTTCATCATCTACGATGCCGTGCACTCTCGTCCTTTCCATCTGTACGCTCACGTCCTTTCGTCCACATGCCCTTCCTGCCACGCAGCACCGCGTGCTGGCCGCCCGCGACAGCGAGGGCGTGCAGCCGCTGTTCTGGGGCTGCACCGACAGCGGCCAGCTCATGTTCGGCAGCGTGGCCGACGACCTGGACGGATGCAACCCCACCGCCGCGCCCTTCCCCTCCGGCACCCTGTTCGCCAGTGAGCGGCACACGGTGGCGTACAGCCCCGGTGCTTACGGCTGGGTGATTGTGGATGATGACTTTCCCGGCCTCATCATGAGCTTCCAGCGCACCAAGGAGGGGGCAGAGGGCTGGCGCAACGTCAAGGCCATTCCGCGCGTCACCAGCAAGGGCGTGGTGTGCGGCGCGGTCTACAAGGTGGCGAGCGCGCAGAACATCGACTCCGCCTAA

**Isoform 4 Protein: (239 AA)**

MVLHMLAVFGRETAHPPVLIESEEALKKTHAPMLTDEEQAAEDAACAKVIDTVFNAAKPASRHKQLLGKGSGYLYEASPYCSYAERDGVHVIFTGEVGEWPGIDVVSSAHDAFVRNEPPLEANDAAWLLDFYGTFGRGASESTTQRALECLARVKGTFAFIIYDAVHSRPFHLYAHVLSSTCPSCHAAPRAGRPRQRGRAAAVLGLHRQRPAHVRQRGRRPGRMQPHRRALPLRHPVRQ
